# TGF-β-activated circRYK drives glioblastoma progression by increasing VLDLR mRNA expression and stability in a ceRNA- and RBP-dependent manner

**DOI:** 10.1186/s13046-024-03000-3

**Published:** 2024-03-08

**Authors:** Yuhang Wang, Binbin Wang, Wenping Cao, Xiupeng Xu

**Affiliations:** Department of Neurosurgery, The First Affiliated Hospital of Nanjing Medical University, Nanjing, Jiangsu Province 210000 China

**Keywords:** Glioblastoma, TGF-β, circRNA, VLDLR

## Abstract

**Background:**

The TGF-β signalling pathway is intricately associated with the progression of glioblastoma (GBM). The objective of this study was to examine the role of circRNAs in the TGF-β signalling pathway.

**Methods:**

In our research, we used transcriptome analysis to search for circRNAs that were activated by TGF-β. After confirming the expression pattern of the selected circRYK, we carried out in vitro and in vivo cell function assays. The underlying mechanisms were analysed via RNA pull-down, luciferase reporter, and RNA immunoprecipitation assays.

**Results:**

CircRYK expression was markedly elevated in GBM, and this phenotype was strongly associated with a poor prognosis. Functionally, circRYK promotes epithelial-mesenchymal transition and GSC maintenance in GBM. Mechanistically, circRYK sponges miR-330-5p and promotes the expression of the oncogene VLDLR. In addition, circRYK could enhance the stability of VLDLR mRNA via the RNA-binding protein HuR.

**Conclusion:**

Our findings show that TGF-β promotes epithelial-mesenchymal transition and GSC maintenance in GBM through the circRYK-VLDLR axis, which may provide a new therapeutic target for the treatment of GBM.

**Supplementary Information:**

The online version contains supplementary material available at 10.1186/s13046-024-03000-3.

## Introduction

Glioblastoma (GBM) is among the most lethal and resistant types of malignant solid tumours [1, 2]. Although improvements have been made in the treatment of GBM [3], favourable results are frequently not observed; as the illness worsens, individuals with these tumours typically have a bleak outlook and poor quality of life [4, 5]. Accumulating evidence indicates that glioblastoma stem-like cells (GSCs) play a role in promoting tumour formation, resistance to radiation and chemotherapy, and disease recurrence [6, 7].However, the specific mechanism responsible for the maintenance of GSC tumorigenicity remains unclear [8–10].

Transforming growth factor beta (TGF-β) is a multifunctional cytokine that plays a vital role in regulating the progression of glioblastoma (GBM) and the ability of glioblastoma stem cells (GSCs) to regenerate themselves [11, 12]. Glioma patients with high TGF-β activity have a worse prognosis than do those with low activity. Research has shown that TGF-β1 stimulates the proliferation of glioma-initiating cells (GICs) by activating LIF or Sox2 [13]. Suppression of TGF-β function reduces the number of CD44^high^/ID1^high^ GICs by inhibiting the activities of ID1 and ID3 [14, 15].

Circular RNA (circRNA) is a distinct kind of noncoding RNA molecule [16]. Circular RNA (circRNA) molecules exhibit a closed circular loop that stays intact even in the presence of RNA exonucleases [17]. CircRNAs lack distinct 5′ and 3′ ends, which are features of linear RNAs [18]. CircRNAs have several functions; for example, they can act as transcriptional regulators, RNA-binding proteins (RBPs), and microRNA (miRNA) sponges [19, 20]. Recent studies have revealed that circRNAs inhibit the malignant progression of breast, liver, and gastric cancers by sponging miRNAs or binding RBPs [21, 22]. However, many underlying mechanisms that in glioblastoma have not been identified.

Our recent study showed that TGF-β-activated circRYK expression is elevated and associated with glioma prognosis. Furthermore, we investigated the function and mechanism of circRYK in glioblastoma. Functionally, the downregulation of circRYK reduced GBM cell migration, invasion, and GSC maintenance. The circRYK molecule may function as a molecular sponge for miR-330-5p, leading to an increase in the expression of the VLDLR oncogene and advancing the progression of GBM. Moreover, we found that circRYK promotes the growth of GBM by enhancing the production and stability of VLDLR mRNA via RNA-binding proteins. Furthermore, the expression of circRYK is closely associated with poor prognosis in GBM patients. The findings indicate that circRYK might function as a prognostic biomarker and a promising target for therapeutic intervention in GBM.

## Materials and methods

### Clinical specimens

From the Jiangsu Provincial People’s Hospital, surgical resection was used to retrieve specimens from 40 glioma patients. As negative controls, normal brain tissue samples (NBTs) from 10 individuals with cerebrovascular abnormalities, severe traumatic brain injury, and ongoing craniotomy decompression were also used. Following a liquid nitrogen freeze, samples were surgically removed and preserved. For the purpose of identifying glioma samples, skilled pathologists employed World Health Organization (WHO) diagnostic standards. Our study plan was authorized by the ethical council of Nanjing Medical University, and informed permission was acquired from the patients and their families for each tissue sample. Specific patient data are included in Supplementary Table 1.

### Cell lines and cell culture

The Chinese Academy of Sciences Cell Bank (Shanghai, China) was used to get the glioma cell lines U87, U251, U118, LN229, and T98. After being created from surgical GBM specimens, the primary GBM cell line (pGBM-1) was harbored in a DMEM medium with 10% FBS [23]. Supplementary Table 2 outlines the details of pGBM-1. Normal human astrocytes (NHAs) were provided courtesy of the American Type Culture Collection (ATCC). For TGF-β assays, glioma cells were treated with 0 ng/ml or 10 ng/ml TGF-β1 for 24 h [24]. Stable cloned cell lines were screened and isolated for subsequent functional studies in vitro and *in vivo.*

### Transfection

The shRNAs, miR-330-5p mimics, miR-330-5p antagomir (100 nmol/L), overexpression plasmids, and their respective negative controls were obtained from GenePharma (Shanghai, China). These molecules were transfected into cells using Lipofectamine 3000 (Invitrogen, Carlsbad, CA, USA) following the instructions provided by the manufacturer.

### Western blot assay

Using RIPA buffer obtained from KeyGEN, multiple clusters of GBM cells were lysed at low temperatures. The protein lysates were then centrifuged at a velocity of 12,000 RPM revolutions per minute. After collecting the supernatant, it was electrophoresed on a polyacrylamide gel that contained 10% SDS. The gel was transferred to a PVDF membrane (Millipore, USA), and 5% skim milk was used to block it. Following that, primary antibodies were added and kept at 4 °C for the whole night [25]. Subsequently, the membranes were incubated with either anti-rabbit IgG or anti-mouse IgG at room temperature for two hours. The gel imaging system was then utilized for exposure (Bio-Rad, USA). Further information on the complete set of antibody data is included in supplementary Table 2.

### Immunohistochemistry

The mouse brain was sliced into sections that were 3.5–4 mm thick, paraffin-embedded, and preserved in 4% paraformaldehyde (BOSTER, Wuhan, China). Slices of the paraffin-fixed, alcohol-hydrated brain tissue were taken out in xylene and stained with HE [26]. After three 5-minute washes in PBS, the samples were stained for five minutes with hematoxylin (Sigma, USA). Eosin (USA, Sigma) was used as a staining agent for a duration of two minutes to facilitate microscopic examination of the purity of the nuclei and cytoplasm. A standard dehydration, sealing, microscope analysis, and photo collecting came next.

### Quantitative RT–PCR

Utilizing TRIzol (Invitrogen), total RNA was isolated from tissues and cells. The mRNA was reverse transcribed using PrimeScript RT Master Mix (TaKaPa) [27]. With 18 S rRNA acting as the internal reference, the relative quantification of circRYK and VLDLR was calculated using the 2^–ΔΔCT^ method [28]. For circRYK and VLDLR, standardization to β-actin was performed, respectively. Each of these responses was executed three times. Thermo Fisher’s StepOnePlus Real-Time PCR System was utilized for the quantitative PCR experiments, and agarose gel electrophoresis (AGE) was utilized to analyze the results. The extra file’s Supplemental Table 2 lists all primer sequences.

### Transwell assay

As previously mentioned, Transwell was utilized to assess the invasion and migration of glioma cells. The two significant modifications were switching to a modified 8 mm pore size culture technique and covering the top chamber with matrigel. After 10%FBS medium and serum-free media were added to Transwell Inserts, transfected cells were added [29]. Cells were fixed after a 24-hour growth phase, crystal violet staining, and storage in 4% paraformaldehyde. The number of cells that could be seen through the perforations was counted after photographs were taken using an Olympus microscope and ImageJ software. For each experiment, each step was repeated three times.

### Wound‑healing assay

Prior to being transferred to the confluence, GBM cells were cultivated in 6-well plates to assess the impact of transfection on each individual GBM cell line. A 200 µL pipette tip was utilized to scrape cell monolayers. Cell movement was recorded utilizing an Olympus microscope (Olympus, Tokyo, Japan) at 0 h, 24 h, and 48 h.

### Immunofluorescence

The cells were seeded into 24-well culture plates, and lentiviral vectors were then used to silence circRYK (shcircRYK) or function as a control (shCONT), fix the cells in 4% paraformaldehyde for 15 min, then permeabilize the cells with 0.3% Triton X-100 for 30 min at 37 °C. The cells were treated with FITC-conjugated secondary antibody and 5% BSA in PBS for one hour at room temperature in the dark, after which they were blocked for thirty minutes with 10% goat serum. Fluorescent Abs were then incubated at 4 °C for an additional night. The cells were then stained with DAPI for 5 min. Pictures were obtained utilizing a confocal microscope (Leica Microsystems) [30].

### Three-dimensional spheroid assay

At a viscosity of 2 × 10^4^ cells/mL and 70% confluence, expanded transfected cells were added to 96-well ultra-low adherence plates (#7007, Costar). The inclusion of matrigel enabled the cells to join to form a multicellular spheroid after 96 h and then after 48 h. Fluorescence microscopy was used to analyze cell motility after that.

### Glioma stem cell viability assay

After being gathered, digested, resuspended, and distributed onto sizable plates, the GSCs were cultured for 48 h at 37 °C with 5% carbon dioxide. GSC cells were harvested and centrifuged at 600 rpm for 5 min to eliminate nonviable cells, and the sample was then analyzed [31]. The supernatant was discarded after adding and suspending 2 mL of trypsin for 5 min. After adding two to three millilitres of medium and resuspending the sample, the supernatant was extracted by repeating centrifugation. The volumes of 1 × 10^3^ cells were estimated after the counting of the cells (1 × 10^3^ cells per well). Each well in the control and experimental groups received 200 µL of media and a volume of 1 × 10^3^ cells, and the procedure was repeated for 6 wells in each group. After 30µL of the reagent had been added and had been stirred for 5 min, the activity of a microplate reader (Thermo Scientific, USA) was measured.

### GSCs sphere formation

Centrifugation was used to digest and extract the serum-containing medium from the GSCs. After that, PBS was used to wash them twice. GSCs were cultured on ultra-low adhesion cell culture plates for a duration of 10 days, with about 1 × 10^3^ cells per well on 6-well plates. The growth of spheres was observed using an Olympus microscope. Subsequently, the rate at which spheres were formed was computed.

### Extreme limiting dilution assay

The GSCs were placed in 96-well plates with varying cell quantities per well and then underwent limiting dilution experiments after appropriate modifications or treatments. Seven days after incubation, the tumor sphere formation in each well was assessed. The extreme limit dilution analysis (ELDA) technique was employed to ascertain the presence of stem cells [18].

### RNA-protein immunoprecipitation (RIP)

The RNA Binding Protein Immunoprecipitation Kit for Magna RIP (Millipore, MA, USA) was employed to execute the RIP experiment. The lysates of U87 and pGBM-1 cells were incubated with beads coated with anti-AgO2, anti-HuR, and IgG antibodies overnight at 4 °C [32]. The RNA complex was then extracted from the beads using an elution method called RNeasy MinElute Cleanup Kit (Qiagen, USA). By reversing the aforesaid RNA complex and then performing qRT‒PCR, the level of RNA enrichment on the probes was identified.

### Dual‑luciferase reporter assay

The luciferase reporter gene was created by introducing the circRYK or VLDLR 3′UTR sequence, which has both the predicted and modified binding sites, into the pGL3 vector. circRYK-wt, circRYK-mut, VLDLR 3′UTR-wt, and VLDLR 3′UTR-mut were some of these constructs. After that, cells were co-transfected with the luciferase reporter gene and either miR-330-5p or miR-NC using Lipofectamine 3000 (Invitrogen, USA). Luciferase activity was measured according to protocol using the dual-luciferase reporter gene assay kit (Vazyme, China) [33].

### RNA pulldown

CircRNA pulldown was carried out using a magnetic RNA-protein pull-down kit from Thermo Fisher, Massachusetts, USA. The sequences ATCGAGTTCTGCTTACATAT-/3bio and AAGTCAGTGTTACTACGGAA-/3bio correspond to the 3’ biotin-labeled probes (RiboBio China) designed to target the junction position of circRYK. The RNA extracted from U87 cells that have an increased expression of circRYK (1.5 × 10^7^) was subjected to individual treatment with 100 nmol of probes specific to circRNA and non-specific probes (NC probes) at a temperature of 70 °C for 5 min. The RNA mixture was then let to sit in the presence of the streptavidin magnetic beads for 30 min at room temperature. The aforementioned beads were subjected to incubation with a total of 100 proteins at a temperature of 4 °C while being rotated. Prior to this, any unbound RNA was eliminated using a solution containing 20 mM Tris. Following a duration of one and a half hours, we eliminated the proteins that were not connected using a 50 µL elution solution and gathered the RNA-binding proteins. Finally, silver stain and mass spectrometry were performed on the supernatant (OE Biotechnology, Shanghai, China). To ascertain how VLDLR 3’UTR interacts with HuR, the same approach was applied to VLDLR 3’UTR biotin-labeled probes (RiboBio, China) [34].

### Animal studies

The Nanjing Medical University’s experimental guidelines and the Ministry of Health’s authorized experimental design were followed in all animal tests. IACUC-2,205,076 was the ethical review number, and it was authorized. An orthotopic carcinogenesis model was established in nude mice with the pGBM-1 cell line. Using lentiviruses with the shcircRYK and shCONT sequences, pGBM-1 cells were transplanted into two randomly chosen nude mice. A total of 500,000 pGBM-1 cells were injected into the brain using stereotactic techniques to measure the size of the tumors inside the brain, and they were often placed in the imaging system. Before each trial, mice were anesthetized and injected intraperitoneally with 50 mg/ml of fluorescein potassium salt. After that, they were put in an IVIS imaging system for 10–120 s. Using the Living Images program (Calliper Life Sciences), the fluorescence intensity in the brain area where the tumor first appeared was tracked.

### Statistical analysis

For each experiment, each step was carried out three times. The statistical analysis was performed using SPSS 17.0 (SPSS, USA), and the data were transferred to GraphPad Prism 8 (La Jolla, USA) for visualization. A bioinformatics study was performed using R software (version 4.0). A two-tailed Student’s t-test was performed to determine if there were statistically significant differences between the groups. We used Pearson correlation to analyze the relationship between circRYK and miR-330-5p. The Kaplan-Meier method was applied to calculate the overall survival rate. Prior performing t-tests or ANOVA, tests for normality were conducted, and the data met the criteria for a normal distribution. *P* < 0.05 was considered to indicate statistical significance.

## Results

### CircRYK is upregulated by TGF-β1 in GBM

**Fig. 1:**
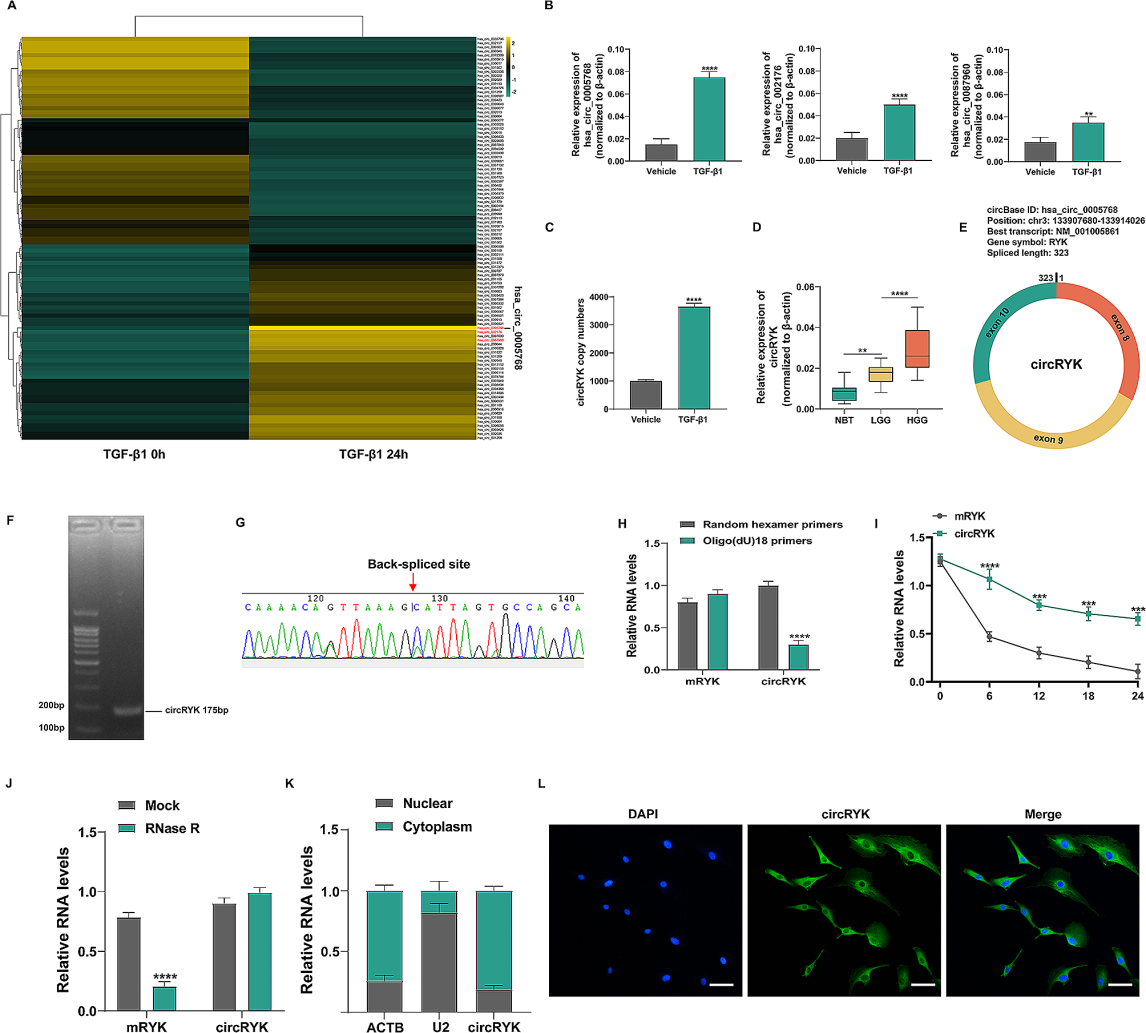
CircRYK is upregulated by TGF-β1 in GBM. (**A**) Heatmap of all differentially expressed circRNAs after treatment of primary cells with 0 ng/ml or 10 ng/ml TGF-β1 for 24 h. (**B**) The expression levels of the three most highly expressed circRNAs were confirmed via qRT‒PCR. (**C**) qRT‒PCR was utilized to confirm the copy numbers of circRYK. (**D**) The expression of circRYK was analysed in ten normal brain tissues (NBTs), twenty low-grade gliomas (LGGs), and twenty high-grade gliomas (HGGs) by qRT‒PCR. (**E**) The splicing sequence and genomic position of circRYK are shown schematically. (**F**) Agarose gel electrophoresis was used to confirm the existence of the circRYK primer (175 bp). The “head to tail” splicing locations of circRYK are shown by the arrow. (**G**) A schematic representation of the structure of circRYK was generated via Sanger sequencing. (**H**) The reverse transcription studies employed random hexamers or oligo (dT)18 primers. qRT‒PCR was utilized to measure the relative RNA levels, and random hexamer primers were used as a standard. (**I**) After actinomycin D treatment, qRT‒PCR was used to calculate the relative quantities of RNA in U87 cells at specific time points. (**J**) The total RNA levels in U87 cells were evaluated via qRT‒PCR after treatment with RNase R or a control treatment. (**K**) The cellular localization of circRYK was examined utilizing cellular RNA fractionation methods. (**L**) FISH was utilized to analyze the cellular localization of circRYK. CircRYK is shown in green. Nuclei were stained with DAPI. Scale bar, 100 μm. Each experiment was performed three times, and the results are displayed as the mean ± SD (**P < 0.01, ***P < 0.001, ****P < 0.0001)

We conducted an RNA-seq study on GBM primary cells treated with 0 ng/ml or 10 ng/ml TGF-β1 for 24 h to investigate TGF-β1-regulated circRNAs. In the TGF-β1-treated group, 49 circRNAs were downregulated, and 51 differentially expressed circRNAs were upregulated (Fig. 1A). According to the RNA-seq findings, hsa_circ_0005768, hsa_circ_002176, and hsa_circ_0087960 had the most significant increases in expression among the 51 circular RNAs. qRT‒PCR was performed to further validate the expression levels of the three circRNAs in primary cells after TGF-β1 treatment, and we discovered that the expression level of hsa_circ_0005768 (circRNA RYK) was the most substantially elevated (Fig. 1B), and qRT‒PCR was used to determine the copy numbers of circRYK (Fig. 1C). The expression level of circRYK was further verified by qRT‒PCR in various grades of glioma, and we discovered that the expression level increased as the glioma grade increased (Fig. 1D). Circularization of exons 8 to 10 of the RYK gene produces circRYK, a novel circRNA that has not been previously studied in gliomas, according to a bioinformatics study. After splicing, the length of mature circRYK is 323 bp (Fig. 1E). To assess the expression of circRYK in glioma tissue, we first developed a special PCR primer for circRYK. Agar gel electrophoresis and Sanger sequencing were used to confirm the sequence and circular structure (Fig. 1F-G). We subsequently performed a variety of experiments to confirm that circRYK has the characteristics of a circular RNA. Initially, we performed reverse transcription on total RNA extracted from U87 cells using either oligo(dT)18 primers or random hexamers. Compared to the results produced with the random hexamer primer, the expression level of circRYK employing the oligo(dT)18 primer was significantly lower. However, mature RYK mRNA (mRYK) expression remained unaltered, and the findings showed that circRYK has no poly(-A)-tail (Fig. 1H). By comparing the degradation rates of circRYK and mRYK in the pGBM-1 cell line after treatment with the transcription inhibitor actinomycin D, we also discovered that circRYK was more stable than mRYK was (Fig. 1I). RNase R was utilized to break down total RNA since earlier studies have shown that circRNAs are more stable than linear RNAs and resistant to RNase R digestion. The results indicated that circRYK exhibited greater resistance to destruction by RNase R than linear RYK mRNA (Fig. 1J). Our investigation revealed that the cytoplasm had the largest portion of circRYK in GBM cells, as determined using cellular RNA fractionation (Fig. 1K), and these findings were also confirmed using FISH (Fig. 1L). These findings indicated that circRYK is a stable circulating transcript.

### circRYK induces GBM cell epithelial-mesenchymal transition and GSC maintenance in vitro

**Fig. 2:**
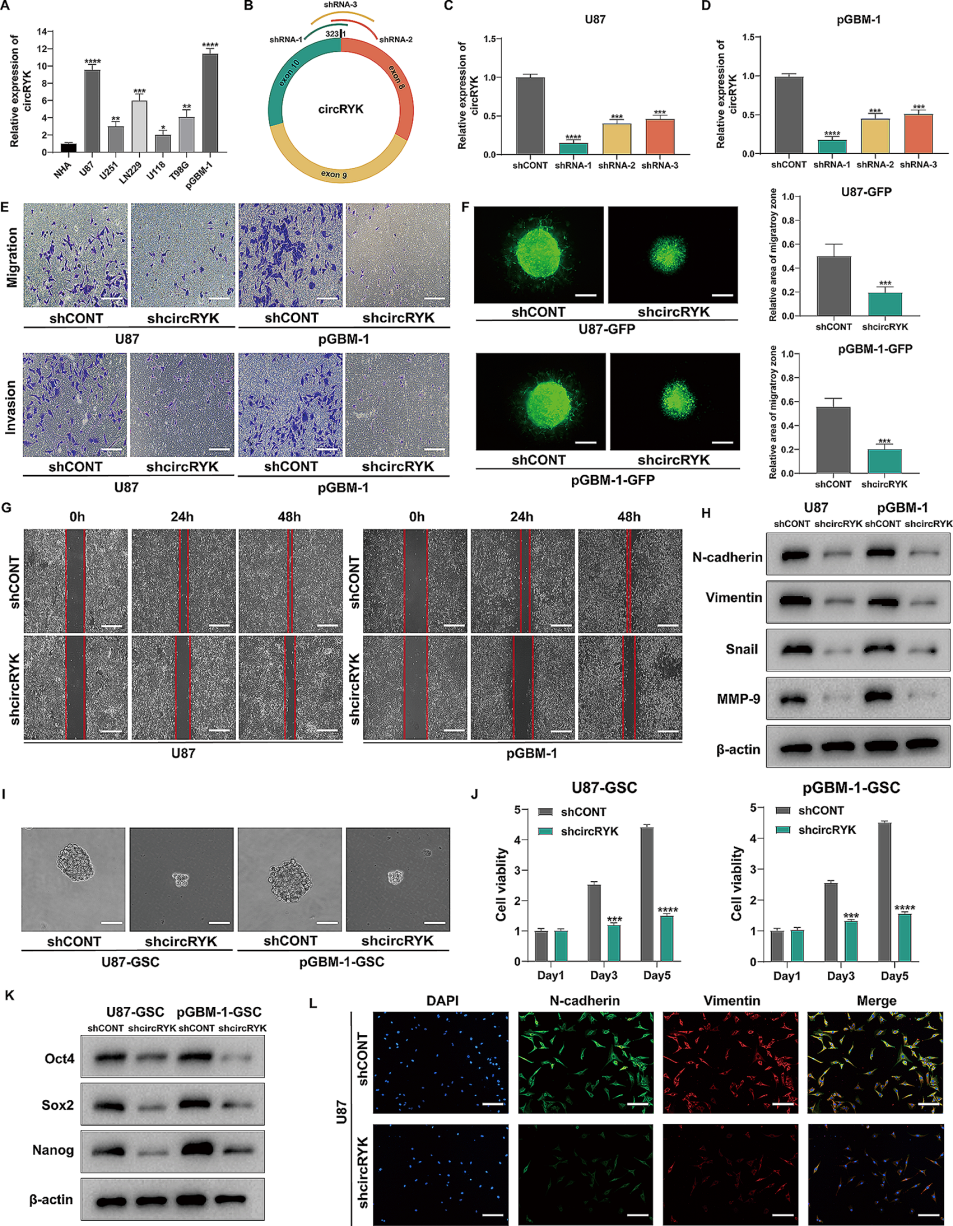
CircRYK induces GBM cell epithelial-mesenchymal transition and GSC maintenance in vitro. (**A**) qRT‒PCR was utilized to examine the levels of circRYK expression in GBM cells and NHAs. (**B**) At splice junctions, designated shRNAs for circRYK are illustrated schematically. (**C-D**) qRT‒PCR was used to evaluate the expression of circRYK in GBM cells transfected with circRYK-targeting shRNA. (**E**) Transwell assays for cell EMT research following glioma cell transfection with expression vectors or shRNA. (**F**) The invasion of U87-GFP and pGBM-1-GFP tumor spheres was observed using three-dimensional spheroid experiments. The quantitative findings are shown together with representative photographs taken three days after plating. (**G**) The migration of GBM cells was examined via a wound healing assay. (**H**) After transfection, the expression of N-cadherin, Vimentin, Snail, and MMP-9 in GBM cells was analysed via Western blotting. (**I**) The stemness of GSCs following transfection was evaluated via a clonogenic trial. (**J**) The CellTiter-Glo assay was used to assess the effects on GBM-GSC cells. (**K**) Western blots were performed to assess the expression of Sox2, Oct4, and Nanog in GBM-GSC cells after transfection. (**L**) Immunofluorescence staining was applied to determine the expression of N-cadherin and Vimentin in treated U87 cells. Scale bar, 100 μm. Each experiment was performed three times, and the results are displayed as the mean ± SD. CONT, control (*P < 0.05, **P < 0.01, ***P < 0.001, ****P < 0.0001)

We used quantitative reverse transcription polymerase chain reaction (qRT‒PCR) to investigate the expression of circRYK in several cell lines, including the pGBM-1 human glioblastoma primary cell line, U87, U118, U251, T98, LN229 and normal human astrocytes (NHAs). Compared to that in other cell lines, circRYK expression was higher in GBM cells (Fig. 2A). To conduct the subsequent studies, we used pGBM-1 and U87 cells. First, we transfected three distinct lentiviral vectors encoding shRNAs that specifically targeted circRYK, designated shRNA-1, shRNA-2, and shRNA-3, to conduct loss-of-function investigations (Fig. 2B). We selected shRNA-1 (shcircRYK) for further investigation based on qRT‒PCR data demonstrating the efficacy of circRYK knockdown (Fig. 2C-D). The inhibition of circRYK expression resulted in a reduction in the migration and invasion of GBM cells, as shown by the Transwell and wound healing experiment (Fig. 2E, G and sFig. 1 A-D).We demonstrated that inhibiting circRYK significantly decreased the invasive ability of GBM cells, as shown by a three-dimensional spheroid experiment (Fig. 2F). The levels of EMT markers (N-cadherin, Vimentin, Snail, and MMP-9) were considerably lower when circRYK was silenced (Fig. 2H). These results were further verified by immunofluorescence analysis (Fig. 2L and sFig. 1 K).

Subsequently, we further verified whether circRYK had any effect on the maintenance of GSCs (U87-GSCs and pGBM-1-GSCs). Our study revealed that the viability and proliferation capacity of GSCs were diminished upon the knockout of circRYK (Fig. 2I-J and sFig. 1E-J). Subsequently, the levels of the pluripotency factors Sox2, Oct4, and Nanog were assessed via Western blotting (Fig. 2K).

### Knockdown of circRYK inhibits GBM growth in vivo

**Fig. 3:**
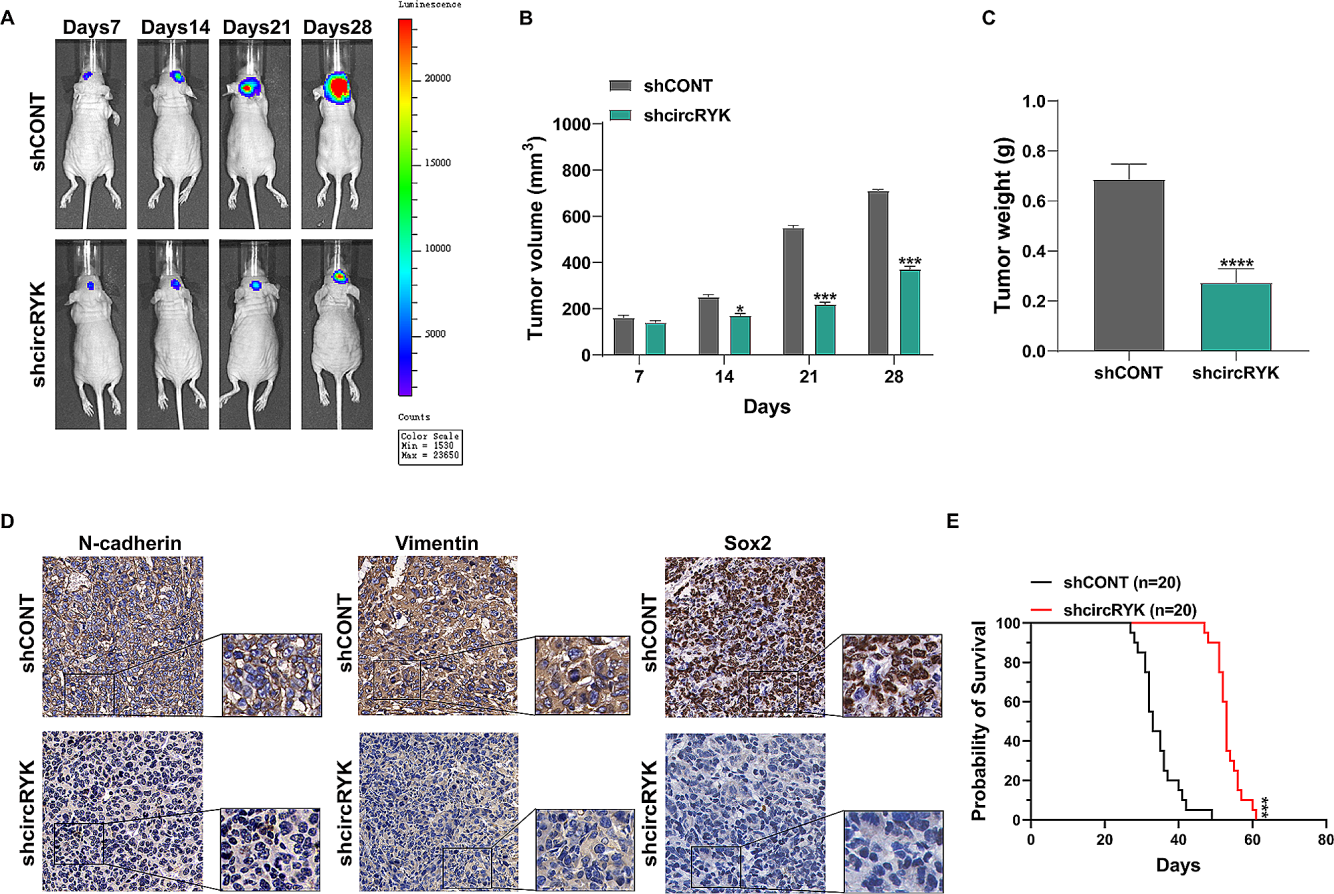
Knockdown of circRYK inhibits GBM growth in vivo. (**A-C**) Bioluminescence pictures of brain tumors were taken weekly in nude mice after the precise implantation of glioma cells. Each group consisted of 10 animals. (**D**) Immunohistochemistry was used to evaluate the shCONT and shcircRYK xenograft tumours. (**E**) Kaplan‒Meier analysis of survival data showed that shcircRYK mice survived shCONT. Scale bar, 100 μm. Each experiment was performed three times, and the results are displayed as the mean ± SD (*P < 0.05, ***P < 0.001, ****P < 0.0001)

To gain more insight into the impact of circRYK on glioma cells in living organisms, we established a model of intracranial xenograft tumours. pGBM-1 cells transfected with either shCONT or shcircRYK were stereotaxically injected into the brain. We implanted pGBM-1 cells intracranially in nude mice and conducted weekly bioluminescence imaging to monitor tumor growth. Suppressing circRYK significantly decelerated the progression of brain tumors (Fig. 3A-C). Immunohistochemistry examination validated the in vitro findings by showing reduced N-cadherin, Vimentin, and Sox2 levels in samples from shcircRYK pGBM-1 cells.(Fig. 3D). The Kaplan‒Meier survival analysis showed that mice injected with shcircRYK had a longer survival time than those injected with shCONT (Fig. 3E).

### Knockdown of circRYK reverses TGF-β1-induced epithelial-mesenchymal transition and GSC maintenance

**Fig. 4:**
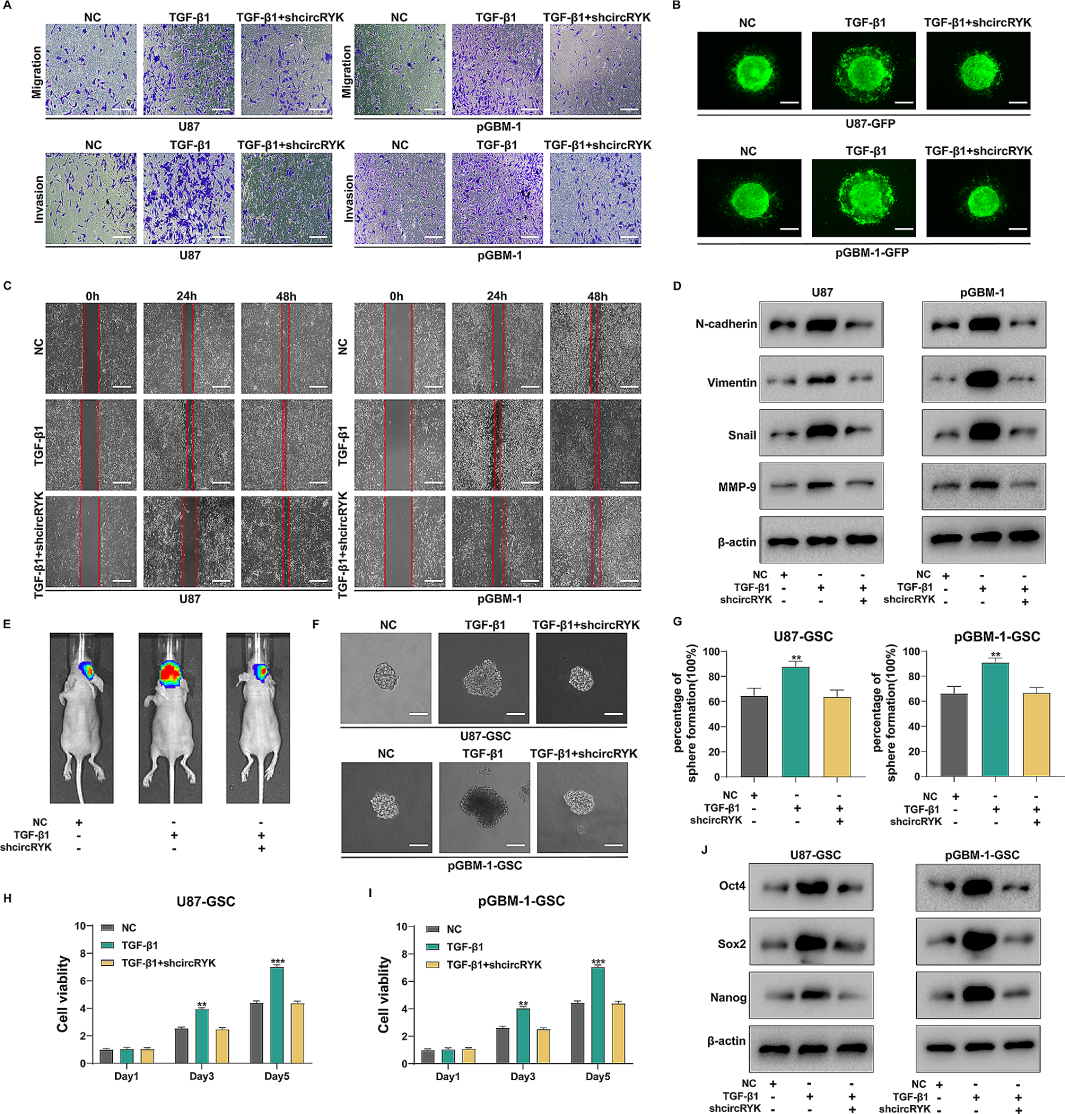
Knockdown of circRYK reverses TGF-β1-induced epithelial-mesenchymal transition and GSC maintenance. (**A**) Transwell assays showed that the promoting effect of TGF-β1 treatment was reversed by knockdown of circRYK in GBM cells. (**B**) A three-dimensional spheroid test demonstrated that reducing the expression of circRYK in U87-GFP and pGBM-1-GFP cells counteracted the enhancing effect of TGF-β1 treatment. (**C**) A wound healing assay was conducted to verify the effect of simultaneous circRYK knockdown and treatment with TGF-β1 on GBM cell migration. (**D**) Western blotting was employed to evaluate the expression of related markers after simultaneous knockdown of circRYK and TGF-β1 treatment in GBM cells. (**E**) Bioluminescence imaging was used to track the effect of shcircRYK transfection and TGF-β1 treatment on intracranial tumour size. (**F-I**) Clonogenicity and CellTiter-Glo assays demonstrated that knockdown of circRYK reversed the effects of GSC maintenance after TGF-β1 treatment. (**J**) Western blotting was employed to demonstrate that knockdown of circRYK inhibited the increase in the expression of related proteins after TGF-β1 treatment. Scale bar, 100 μm. Each experiment was performed thrice, and the results are displayed as the mean ± SD. NC, negative control (**P < 0.01, ***P < 0.001)

To determine whether TGF-1 regulates circRYK, we incubated GBM cells with 10 ng/ml TGF-β1 and shcircRYK plasmid. First, we discovered that TGF-β1 may promote the migration and invasion of GBM cells, which were reversed by TGF-β1 combined with shcircRYK utilizing Transwell, three-dimensional spheroid, and wound healing experiments (Fig. 4A-C and sFig. 2B-G). The interaction between TGF-β1 and circRYK in EMT was further confirmed via the expression of markers in GBM cells by Western blotting (Fig. 4D). Furthermore, we injected pGBM-1 cells treated with TGF-β1 alone or in combination with shcircRYK into the brains of nude mice and found that shcircRYK reversed the tumour growth effect of TGF-β1 (Fig. 4E). Then, with the treated GBM-GSCs, we conducted clonogenic, CellTiter-Glo, direct cell count, and extreme limiting dilution tests. Similarly, the impact of TGF-β1 on GSC maintenance was reversed by circRYK knockdown (Fig. 4F-I and sFig. 2 H-K), and Western blotting also confirmed this conclusion (Fig. 4J).

### CircRYK acts as a miRNA sponge of miR-330-5p to upregulate VLDLR expression

**Fig. 5:**
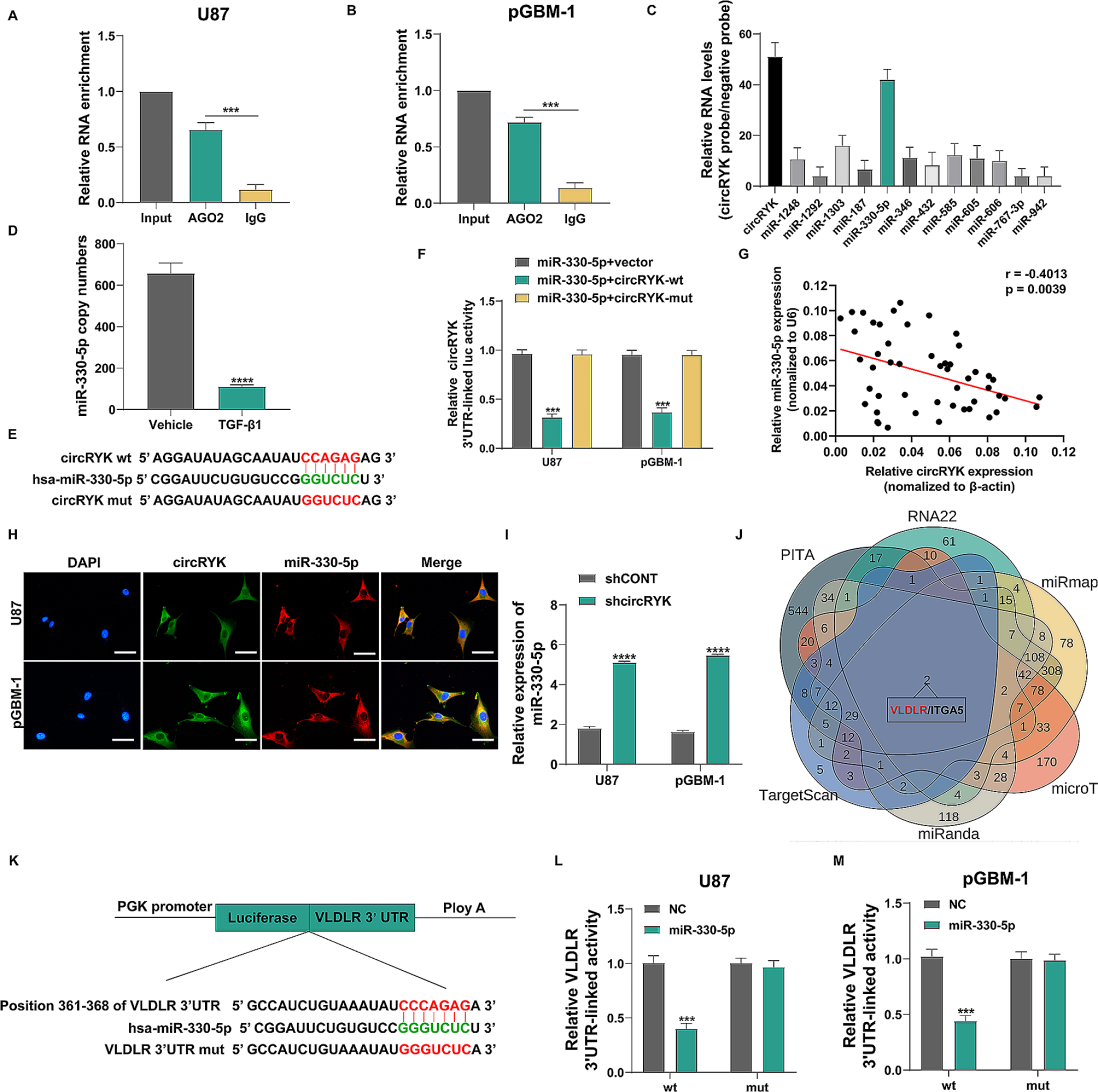
CircRYK acts as a miRNA sponge of miR-330-5p to upregulate VLDLR expression. (**A-B**) RIP and qRT‒PCR methods were used to assess the binding of circRYK to the AGO2 protein. (**C**) U87 cells that overexpress circRYK were mixed with a circRYK-specific probe and a negative control (NC) probe to perform the circRIP experiment. The possible miRNAs linked with circRYK were investigated using qRT‒PCR assays.(**D**) qRT‒PCR was used to confirm the copy numbers of miR-330-5p. (**E**) Potential binding site sequences between the wild-type (wt) and mutant-type (mut) variants of circRYK and miR-330-5p. (**F**) Experiments using dual-luciferase reporter assays were conducted to determine the association between circRYK and miR-330-5p. (**G**) With Pearson’s correlation, the relationship between the expression levels of circRYK and miR-330-5p was examined (*r*=-0.4013, *p* = 0.0039). (**H**) The colocalization of circRYK and miR-330-5p in transfected GBM cells was investigated using FISH. (**I**) qRT‒PCR was utilized to confirm the expression of miR-330-5p in treated GBM cells. (**J**) The biological targets of miR-330-5p may include two mRNAs, as shown by a Venn diagram. (**K**) Conceptual representation of the binding sites between the wild-type (wt) or mutant (mut) VLDLR 3’UTR and miR-330-5p. (**L-M**) A luciferase reporter assay in GBM cells was utilized to confirm the functional connection between miR-330-5p and VLDLR. Scale bar, 100 μm. Each experiment was performed thrice, and the results are displayed as the mean ± SD (***P < 0.001)

According to previous studies, circRNAs mainly serve as sponges for miRNAs to control gene expression [35, 36]. We tested the hypothesis that circRYK acts as a miRNA sponge in glioma cells using RNA immunoprecipitation (RIP) tests with an anti-AGO2 antibody. According to the qRT‒PCR data, the anti-AGO2 precipitate significantly increased the degree of circRYK enrichment. On the other hand, the enrichment of the IgG precipitate circRYK was significantly reduced (Fig. 5A-B), revealing that circRYK could bind to AGO2 and miRNAs. The Circular RNA Interactome Database was used to predict prospective miRNA targets of circRYK. The circRIP test data indicated that circRYK and miR-330-5p are possible targets in GBM cells. Specifically, the circRYK-specific probe demonstrated a higher enrichment of circRYK and miR-330-5p compared to other miRNAs (Fig. 5C). Subsequently, we further confirmed the copy numbers of miR-330-5p by qRT‒PCR (Fig. 5D). To provide additional evidence for this finding, we generated dual luciferase reporter vectors containing both wild-type (wt) and mutant (mut) versions of the circRYK sequence. These vectors were designed to include the expected binding sites of miR-330-5p and circRYK (Fig. 5E). The results showed that the introduction of the miR-330-5p mimic into the cells resulted in a substantial reduction in luciferase activity compared to that in the cells that were transfected with the mutant circRYK vector (Fig. 5F). We also performed luciferase reporter gene experiments for other possible miRNAs and found that the results were not significantly different. This further strengthens the confidence that miR-330-5p is downstream of circRYK (sFig. 5A-D). Furthermore, according to Pearson correlation analysis, circRYK and miR-330-5p expression in glioma samples showed a negative correlation (Fig. 5G). Based on the immunofluorescence analysis, circRYK and miR-330-5p colocalized in the cytoplasm (Fig. 5H). CircRYK downregulation significantly increased miR-330-5p expression in U87 and pGBM-1 cells (Fig. 5I). According to the aforementioned investigations, circRYK may serve as a sponge for miR-330-5p in GBM cells. We next used the bioinformatics databases miRanda, TargetScan, PITA, RNA22, miRmap, and microT to confirm that VLDLR is a potential target of miR-330-5p (Fig. 5J). Previous studies have shown that miRNAs bind to the 3′-UTRs of their target mRNAs to inhibit mRNA transcription or induce mRNA degradation [37, 38]. Therefore, we postulated that miR-330-5p binds to the 3’-UTRs of VLDLR to regulate the progression of gliomas (Fig. 5K). A luciferase reporter experiment was used to ascertain whether miR-330-5p can directly bind to the 3′UTR of VLDLR. For this purpose, the miR-330-5p mimic was cotransfected with either the wild-type or mutant VLDLR 3′UTR. We found that after cotransfection of GBM cells with the miR-330-5p mimic, the trend of decreased activity of the miR-330-5p mimic with the mutated binding site was completely attenuated (Fig. 5L-M).

### CircRYK enhances GBM cell EMT and GSC maintenance through the miR-330-5p/VLDLR pathway

**Fig. 6:**
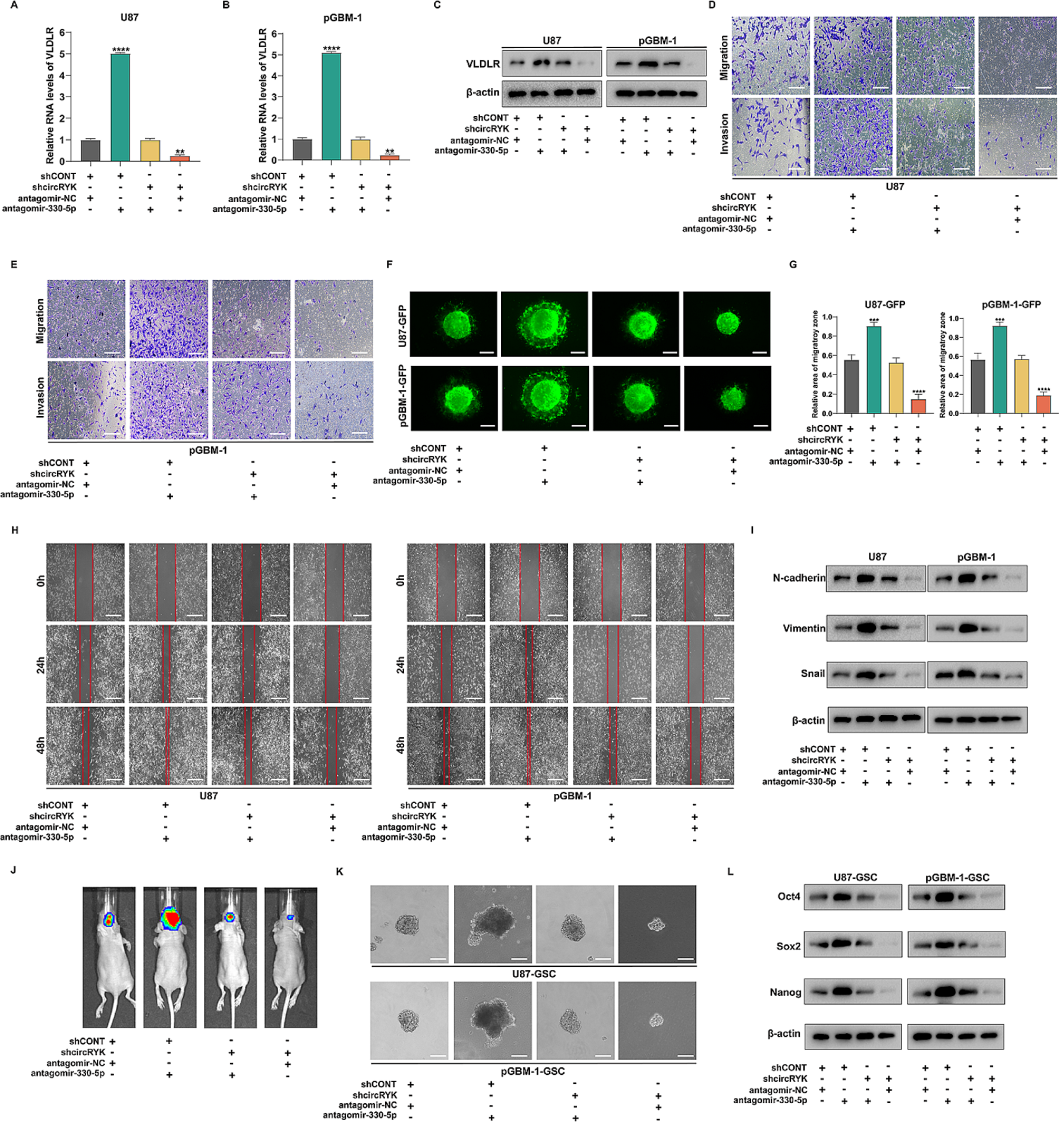
CircRYK enhances GBM cell EMT and GSC maintenance through the miR-330-5p/VLDLR pathway. (**A-B**) qRT‒PCR tests were applied to assess the expression levels of VLDLR in GBM cells after the suppression of miR-330-5p alone or in combination with circRYK. (**C**) In the designated cells, VLDLR was examined using Western blotting, with β-actin serving as a control. (**D-E**) For analysis of the migration and invasion of cells using the Transwell method, GBM cells were genetically modified with shcircRYK, antagomir-330-5p, or a combination of antagomir-330-5p and shcircRYK. (**F-G**) For analysis of the invasion of the cells by three-dimensional spheroid assays, shcircRYK, antagomir-330-5p, or antagomir-330-5p and shcircRYK were transfected into U87-GFP and pGBM-1-GFP cells. (**H**) The wound healing test was utilized to examine the migration of GBM cells that were transfected with shcircRYK, antagomir-330-5p, or both antagomir-330-5p and shcircRYK. (**I**) Western blotting was utilized to investigate the expression of N-cadherin, Vimentin, and Snail in GBM cells after transfection. (**J**) The effect of shcircRYK and antagomir-330-5p transfection, either alone or in combination, on the formation of brain tumours was assessed using bioluminescence imaging. (**K**) The stemness of GSCs after transfection was measured by assessing the proportion of wells exhibiting positive results in a clonogenicity assay. (**L**) The expression of Oct4, Sox2, and Nanog in U87-GSC and pGBM-1-GSC cells was analysed via Western blotting after transfection; β-actin served as the loading control. Scale bar, 100 μm. Each experiment was performed thrice, and the results are displayed as the mean ± SD (**P < 0.01, ****P < 0.0001)

We next investigated the influence of the interaction between circRYK and miR-330-5p on the expression of VLDLR, the aggressiveness of GBM cells, and the maintenance of GSCs. To further confirm the reliability of the ceRNA route, we conducted rescue tests. Suppression of circRYK resulted in a considerable reduction in both VLDLR mRNA and protein levels. Moreover, the reversal of the effect induced by circRYK silencing occurred when we inhibited the expression of miR-330-5p in cells with circRYK knockdown (Fig. 6A-C). In terms of functional studies, Transwell, three-dimensional spheroid assays, and wound healing tests confirmed that circRYK downregulation prevented GBM cells from undergoing EMT and that treatment with a miR-330-5p antagomir reversed the effects of circRYK knockdown (Fig. 6D-I and sFig. 3 A-D). Furthermore, we designed an intracranial tumour model to further confirm that the effect of circRYK knockdown on intracranial tumours was reversed when the miR-330-5p antagomir was transfected (Fig. 6J). Additionally, suppression of miR-330-5p prevented the inhibitory effect of circRYK on GSC maintenance (Fig. 6K-L and sFig. 3E-K). These results demonstrated that circRYK acts as a miR-330-5p sponge, regulating VLDLR expression and promoting the development of gliomas.

### CircRYK interacts with HuR and promotes its cytoplasmic transport

**Fig. 7:**
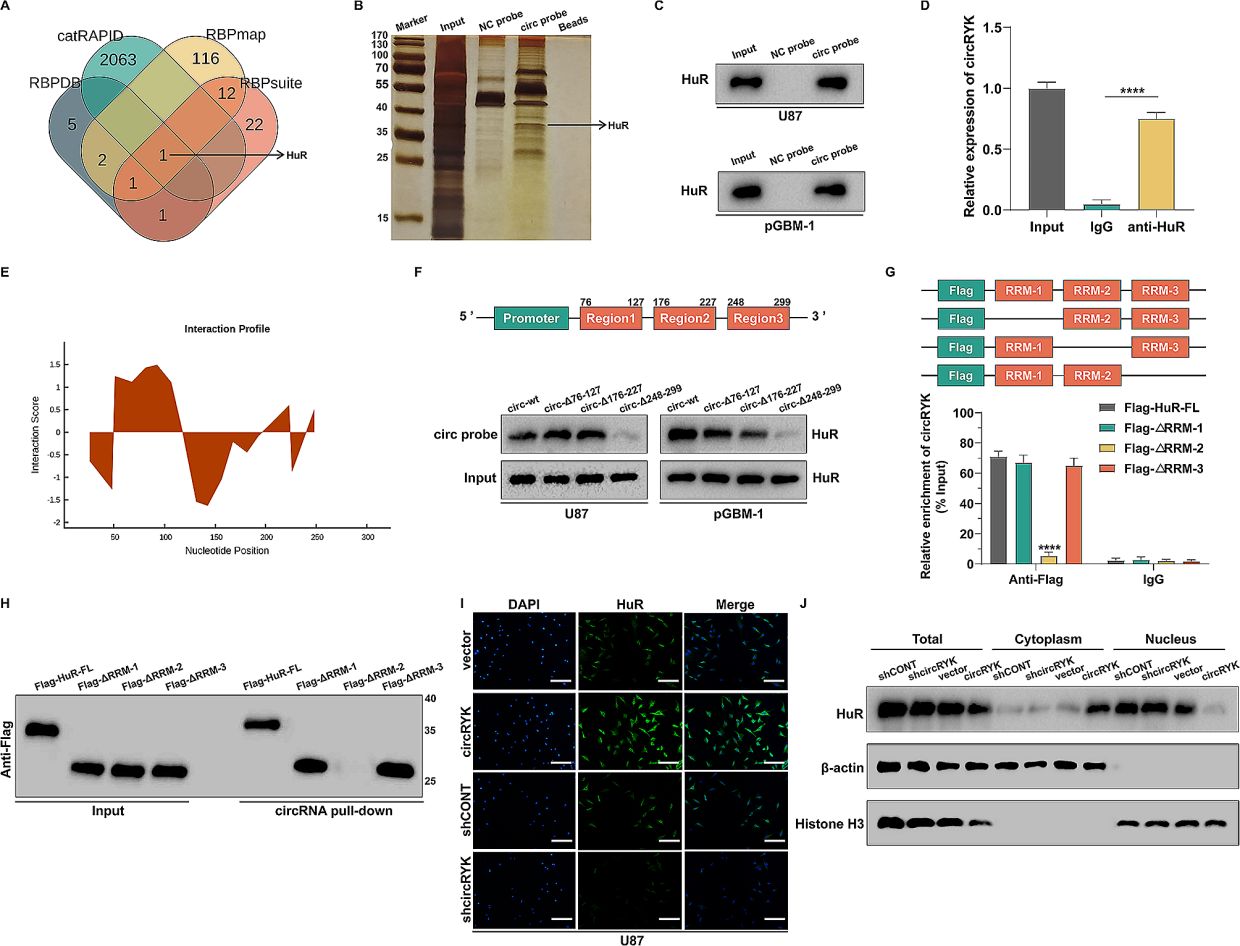
CircRYK interacts with HuR and promotes its cytoplasmic transport. (**A**) The Venn diagram illustrates the potential RNA-binding proteins (RBPs) that circRYK may interact with. (**B**) Silver staining of the circRYK pulldown. (**C**) HuR was confirmed to be enriched in the circRYK probe by Western blot. (**D**) The RIP test findings indicate that the HuR protein formed a precipitate with circRYK in GBM cell lysates. (**E**) The predicted interaction sites between circRYK and HuR. (**F**) The diagram illustrates the truncated portion of the circRYK overexpression plasmid. RNA pull-down was carried out using circRYK-specific probes after GBM cells were transfected with wild-type or truncated circRYK overexpression plasmids. (**G**) The levels of circRYK were determined using qRT‒PCR after treatment of U87 cell lysates with either full-length or truncated versions of Flag-labelled recombinant HuR protein. (**H**) RNA pull-down studies employed circRYK-specific probes with full-length or truncated versions of the Flag-tagged recombinant HuR proteins. (**I**) Immunofluorescence was employed to verify the expression of HuR in U87 cells after the knockdown or overexpression of circRYK. (**J**) Western blotting was employed to determine HuR expression levels in whole lysates or subcellular fractions. The shCONT, shcircRYK, vector, and circRYK overexpression plasmids were transfected into GBM cells. Scale bar, 100 μm. Each experiment was performed three times, and the results are displayed as the mean ± SD (****P < 0.0001)

The surprising finding that circRYK plasmids retained partial cancer-promoting activity when the miR-330-5p binding site was mutated led us to consider the RNA binding proteins (RBP) function of circRYK as the potential effector mechanism. Circular RNAs function as RNA-binding proteins to regulate the expression of genes downstream [39–41]. By bioinformatics analysis, we identified a potential RBP downstream of circRYK (Fig. 7A). To test our hypothesis, we performed RNA pull-down analysis in U87 cells followed by SDS‒PAGE (Fig. 7B). Subsequently, RNA pull-down and RIP assays were used to confirm the interaction between circRYK and HuR. The results indicated that the circRYK probes successfully captured the HuR protein in U87 and pGBM-1 cell lysates (Fig. 7C), and the anti-HuR antibody also precipitated circRYK (Fig. 7D). The three locations where circRYK binds to HuR are 76–127 nt, 176–227 nt, and 248–299 nt, according to the CatRAPID database (Fig. 7E). Next, circRYK plasmids were created with these three truncated sections and then transfected into U87 and pGBM-1 cells. The RNA pull-down assays demonstrated a substantial reduction in the amount of HuR protein captured by circRNA probes when the 248–299 nt region of circRYK was truncated. These findings suggested that circRYK primarily interacts with HuR in this specific area (Fig. 7F). To clarify the specific structural domain of HuR that is involved in its interaction with circRYK, we generated HuR mutants by truncating certain structural domains. RIP tests provided evidence that RNA recognition motif 2 (RRM2) of HuR selectively binds to circRYK (Fig. 7G). Subsequent RNA pull-down experiments conducted in U87 cells revealed that the HuR protein was unable to be captured by circRYK probes when its RRM2 region was truncated (Fig. 7H). HuR, a nucleoplasmic shuttle protein, has several biological roles based on its subcellular location. Our investigation revealed that the overexpression of circRYK facilitated the transport of the HuR protein from the nucleus to the cytoplasm. Conversely, a reduction in circRYK resulted in a decrease in the cytoplasmic concentration of HuR (Fig. 7J). Immunofluorescence consistently showed a direct relationship between the expression of circRYK and the quantity of HuR in the cytoplasm (Fig. 7I).

### CircRYK stabilizes VLDLR mRNA by interacting with HuR

**Fig. 8:**
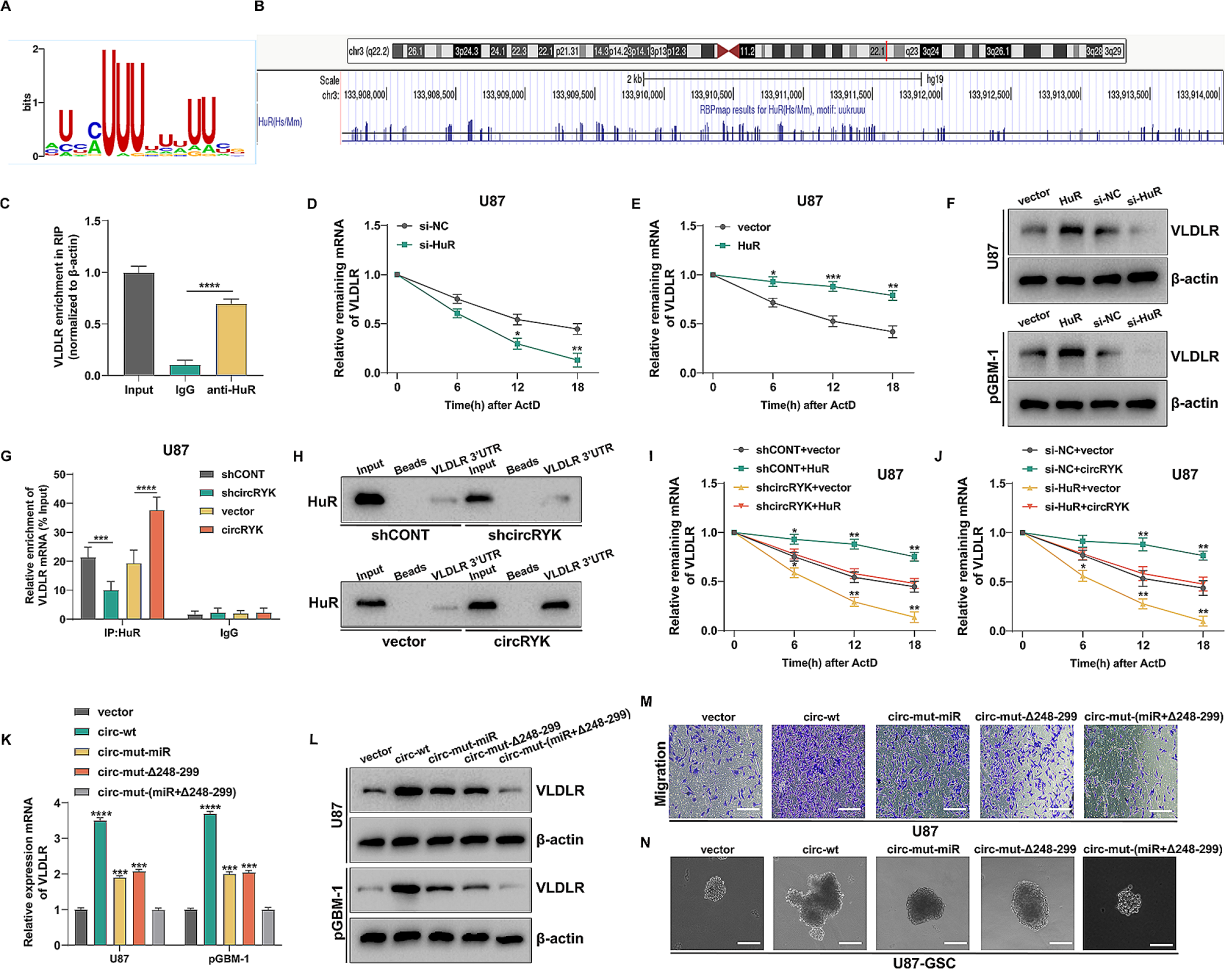
CircRYK stabilizes VLDLR mRNA by interacting with HuR. (**A**) The motif of HuR. (**B**) RBPmap was used to predict the locations of HuR binding sites within the 3’UTR of the VLDLR gene. The vertical blue lines at the bottom display the HuR binding sites in the 3’UTR of VLDLR mRNA. (**C**) The HuR protein interacts with VLDLR mRNA, as shown by the RIP assay. (**D-E**) qRT‒PCR was utilized to measure the rate of VLDLR mRNA degradation in HuR-overexpressing or knockdown U87 cells at various time points. (**F**) Western blotting was utilized to determine the expression of the VLDLR protein in GBM cells transfected with si-NC, si-HuR, vector, or HuR overexpression plasmids. (**G**) RIP experiments revealed that HuR and VLDLR mRNA coprecipitated in U87 cells when circRYK was knocked down or overexpressed. (**H**) In GBM cells, RNA pulldown tests using the biotin-labelled VLDLR 3’UTR were carried out. The circRYK overexpression or shcircRYK plasmids were transfected into GBM cells. (**I-J**) Measurement of the rate at which VLDLR mRNA is degraded in U87 cells after transfection with different plasmids or short interfering RNAs. (**K-L**) The level of VLDLR in GBM cells was assessed after transfection with circRYK overexpression plasmids containing both wild-type and mutant variants. circ-mut-miR denoted a mutation in the miR-330-5p binding region, whereas circ-mut-Δ248-299 denoted a truncated version of the circRYK sequence from position 248 to 299. The term “circ-mut-(miR + Δ248–299)” was used to refer to both the mutant miRNA binding site and the truncated section of the sequence. (**M-N**) Transwell and clonogenicity assays were employed to verify GBM progression in U87 and U87-GSC cells under different treatment conditions. Scale bar, 100 μm. Each experiment was performed three times, and the results are displayed as the mean ± SD (*P < 0.05, **P < 0.01, ***P < 0.001, ****P < 0.0001)

The main role of cytoplasmic HuR is to augment the stability of certain target mRNAs by binding to U- or AU-rich RNA sequences (AREs) found in the 3’-UTR of the mRNAs [42, 43]. The VLDLR mRNA has a lengthy 3’UTR that is rich in U and AU nucleotides and contains many putative HuR-binding motifs (Fig. 8A-B). Compared to the results in the IgG group, the RIP tests showed a substantial increase in VLDLR mRNA in the anti-HuR group (Fig. 8C). In addition, experiments using actinomycin D in GBM cells demonstrated that suppressing HuR resulted in decreased amounts of VLDLR mRNA and a shorter lifespan of its transcript relative to those in the control group. Conversely, overexpression of HuR led to the opposite effects (Fig. 8D-F and sFig. 4A-B). We utilized RIP assays in GBM cells to explore the potential impacts of circRYK on the interaction between HuR and VLDLR. Our findings indicate that reducing the expression of circRYK results in a reduction in the binding of VLDLR mRNA to HuR. Conversely, overexpressing circRYK led to an increase in the amount of VLDLR mRNA bound by HuR (Fig. 8G and sFig. 4 C). RNA pulldown assays consistently revealed that altering the amount of circRYK led to a corresponding change in the amount of HuR protein bound to the 3’ UTR of VLDLR mRNA (Fig. 8H). Rescue experiments revealed that the reduction in the stability of VLDLR mRNA, which was driven by the inhibition of circRYK in GBM cells, was reversed by the overexpression of HuR. On the other hand, the increase in VLDLR mRNA stability caused by circRYK overexpression was not observed when HuR was suppressed (Fig. 8I-J and sFig. 4D-E). To determine whether circRYK affects the biological function of GBM cells by regulating both HuR and miR-330-5p, we transfected U87 and pGBM-1 cells with circRYK plasmids containing mutant miR-330-5p binding sites (circ-mut-miR), truncated HuR binding areas (circ-mut-Δ248–299), or both mutations (circ-mut-(miR + Δ248–299)). These findings indicated that, compared with the empty vector, circ-mut-miR and circ-mut-Δ248–299 had moderate effects on enhancing VLDLR expression. However, circ-mut-miR + Δ248–299 had little impact on increasing VLDLR expression (Fig. 8K-L). In addition, Transwell and clonogenic experiments demonstrated that circ-mut-miR and circ-mut-Δ248–299 only marginally enhanced EMT and GSC maintenance in GBM cells. Conversely, when circ-mut-miR + Δ248–299 was compared to the empty vector, glioma-promoting activity was almost absent (Fig. 8M-N and sFig. 4 F-M). Collectively, our results indicate that circRYK controls the expression of VLDLR via two synchronized mechanisms: acting as a miR-330-5p sponge and acting as a HuR decoy for RNA-binding proteins.

## Discussion

CircRNAs have been confirmed to be important regulators of cancer [44, 45]. This study characterized circRYK as a critical regulator of EMT, GSC maintenance and tumorigenicity in GBM. Our studies indicate that the activation of TGF-β leads to the promotion of glioma growth via the upregulation of VLDLR mRNA expression and stability. This process is mediated by circRYK, which acts as a competing endogenous RNA (ceRNA) and relies on RNA-binding proteins (RBPs). Furthermore, our findings indicate a significant correlation between the expression of circRYK and the progression of glioma. Moreover, glioma patients with elevated levels of circRYK have a poorer prognosis compared to those with lower levels.

This work revealed that circRYK, which is activated by TGF-β, plays a crucial role in regulating both EMT and the maintenance of GSCs. The TGF-β pathway plays a crucial role in the tumour formation of GICs [46–48]. In addition, several anti-TGF-β medications are now undergoing clinical trials. However, inhibiting TGF-β often results in a rapid adaptive reaction that causes resistance to the suppression of TGF-β [49]. Therefore, it is necessary to conduct a deeper exploration of the particular downstream effectors of the TGF-β pathway [50].

Thus far, the majority of circRNA research has shown that circRNAs carry out their biological tasks via three primary mechanisms: acting as ceRNAs, binding to RBPs, and regulating translation [51]. Our research findings confirmed that circRYK and VLDLR have a common microRNA response element (MRE) in miR-330-5p, establishing a circRYK/miR-330-5p/VLDLR axis. Remarkably, our findings indicate that even circRYK overexpression plasmids harbouring mutations in the miR-330-5p binding region exhibited a partial increase in VLDLR expression and facilitate the progression of GBM, although to a lesser extent than wild-type circRYK plasmids. Based on these data, we hypothesized that circRYK may control the expression of VLDLR via other mechanisms. RBPs play a crucial role in regulating transcription and translation processes. Furthermore, they have been shown to participate in the control of subsequent genes via their interaction with circRNAs. In recent years, circRNAs with dual-faceted regulatory mechanisms, including both ceRNAs and RBPs have been discovered [52]. For instance, circPPFIA1s suppresses the spread of colorectal cancer to the liver by affecting the miR-155-5p/CDX1 and HuR/RAB36 pathways [53]. Our investigation revealed a physical interaction between circRYK and the HuR protein. HuR, a conserved regulator of mRNA stability, has several sites for posttranslational modifications [54]. These changes facilitate its transportation from the nucleus to the cytoplasm [55]. The HuR protein is abundantly produced in the majority of cancerous cells [56]. Notably, the accumulation of HuR in the cytoplasm of esophageal cancer, non-small cell lung cancer, and meningioma cells is indicative of a poor prognosis [42]. Thus, it is hypothesized that the capacity of HuR to increase mRNA stability depends on its ability to migrate into the cytoplasm. Chen et al. discovered that the circAGO2 molecule, which is present at high levels, interacts with HuR and facilitates the translocation of HuR from the nucleus to the cytoplasm [57].However, the role of HuR in the development and progression of GBM is still unclear. This study demonstrated that circRYK promotes the cytoplasmic accumulation of HuR, which in turn increases the stability of VLDLR mRNA.

The very-low-density lipoprotein receptor (VLDLR) is a member of the low-density lipoprotein receptor (LDLR) superfamily [58]. Abnormal VLDLR expression has been linked to the development of many types of cancer [59]. However, studies on the role of the VLDLR in glioma are rare, and the underlying carcinogenic mechanism is still unclear. These findings revealed that circRYK enhances the expression of VLDLR by acting as a sponge for miR-330-5p and recruiting HuR. The presence of this dual regulatory mechanism may significantly contribute to the atypical expression of VLDLR. CircPTN has been reported to promote glioma proliferation and stemness by sponging miR-330-5p [60]. However, our experiments revealed that the expression of circPTN did not change significantly in response to TGF-β induction (sFig. 5E-F). Although circPTN can contribute to the malignant progression of GBM, we focused more on the altered circRNAs under TGF-β induction affecting the malignant progression of GBM. Therefore, we did not include circPTN within the range of TGF-β-induced circRNAs. Nevertheless, the regulatory network among genes is complex, and includes several layers, directions, and interactions. Additional research is required to establish whether circRYK influences the expression of VLDLR via other mechanisms.

We also evaluated whether circRYK overexpression per se was sufficient to induce VLDLR expression and ultimately alter the normal human astrocyte state, thus mimicking the glioblastoma phenotype (sFig. 7 A-F). This result further strengthens the function of circRYK as a biomarker and promising therapeutic target and highlights its potentially significant role in the etiology of glioblastoma.

In conclusion, our study revealed that circRYK promotes the growth of GBM by enhancing the production and stability of VLDLR mRNA via a mechanism involving ceRNA and RBP. Additionally, the expression of circRYK is strongly associated with a poor prognosis in GBM patients. The results suggest that circRYK might serve as a predictive biomarker and a potential target for treatment strategies in GBM.

### Electronic supplementary material

Below is the link to the electronic supplementary material.

Supplementary Material 1

Supplementary Material 2

Supplementary Material 3

Supplementary Material 4

## Data Availability

The datasets utilized and/or analyzed in the present study will be made available by the responsible author in response to a reasonable request.

## References

[1] Alexander BM, Cloughesy TF, Glioblastoma A (2017). J Clin Oncol.

[2] Gusyatiner O, Hegi ME (2018). Glioma epigenetics: from subclassification to novel treatment options. Semin Cancer Biol.

[3] Fan X, Salford LG, Widegren B (2007). Glioma stem cells: evidence and limitation. Semin Cancer Biol.

[4] Xu S, Tang L, Li X, Fan F, Liu Z (2020). Immunotherapy for glioma: current management and future application. Cancer Lett.

[5] Ostrom QT, Bauchet L, Davis FG, Deltour I, Fisher JL, Langer CE, Pekmezci M, Schwartzbaum JA, Turner MC, Walsh KM, Wrensch MR, Barnholtz-Sloan JS (2014). The epidemiology of glioma in adults: a state of the science review. Neuro Oncol.

[6] Boyd NH, Tran AN, Bernstock JD, Etminan T, Jones AB, Gillespie GY, Friedman GK, Hjelmeland AB (2021). Glioma stem cells and their roles within the hypoxic tumor microenvironment. Theranostics.

[7] Suva ML, Tirosh I (2020). The glioma stem cell model in the era of single-cell Genomics. Cancer Cell.

[8] Bexell D, Svensson A, Bengzon J (2013). Stem cell-based therapy for malignant glioma. Cancer Treat Rev.

[9] Shah K (2016). Stem cell-based therapies for tumors in the brain: are we there yet?. Neuro Oncol.

[10] Wang Z, Zhang H, Xu S, Liu Z, Cheng Q (2021). The adaptive transition of glioblastoma stem cells and its implications on treatments. Signal Transduct Target Ther.

[11] Chao M, Liu N, Sun Z, Jiang Y, Jiang T, Xv M, Jia L, Tu Y, Wang L (2020). TGF-beta signaling promotes glioma progression through stabilizing Sox9. Front Immunol.

[12] Joseph JV, Magaut CR, Storevik S, Geraldo LH, Mathivet T, Latif MA, Rudewicz J, Guyon J, Gambaretti M, Haukas F, Trones A, Romo Ystaas LA, Hossain JA, Ninzima S, Cuvellier S, Zhou W, Tomar T, Klink B, Rane L, Irving BK, Marrison J, O’Toole P, Wurdak H, Wang J, Di Z, Birkeland E, Berven FS, Winkler F, Kruyt FAE, Bikfalvi A, Bjerkvig R, Daubon T, Miletic H (2022). TGF-beta promotes microtube formation in glioblastoma through thrombospondin 1. Neuro Oncol.

[13] Peng P, Zhu H, Liu D, Chen Z, Zhang X, Guo Z, Dong M, Wan L, Zhang P, Liu G, Zhang S, Dong F, Hu F, Cheng F, Huang S, Guo D, Zhang B, Yu X, Wan F (2022). TGFBI secreted by tumor-associated macrophages promotes glioblastoma stem cell-driven tumor growth via integrin alphavbeta5-Src-Stat3 signaling. Theranostics.

[14] Ikushima H, Todo T, Ino Y, Takahashi M, Miyazawa K, Miyazono K (2009). Autocrine TGF-beta signaling maintains tumorigenicity of glioma-initiating cells through sry-related HMG-box factors. Cell Stem Cell.

[15] Tritschler I, Gramatzki D, Capper D, Mittelbronn M, Meyermann R, Saharinen J, Wick W, Keski-Oja J, Weller M (2009). Modulation of TGF-beta activity by latent TGF-beta-binding protein 1 in human malignant glioma cells. Int J Cancer.

[16] Huang A, Zheng H, Wu Z, Chen M, Huang Y (2020). Circular RNA-protein interactions: functions, mechanisms, and identification. Theranostics.

[17] Zhou WY, Cai ZR, Liu J, Wang DS, Ju HQ, Xu RH (2020). Circular RNA: metabolism, functions and interactions with proteins. Mol Cancer.

[18] Wang Y, Wang B, Zhou F, Lv K, Xu X, Cao W (2023). CircNDC80 promotes glioblastoma multiforme tumorigenesis via the miR-139-5p/ECE1 pathway. J Transl Med.

[19] Lei M, Zheng G, Ning Q, Zheng J, Dong D (2020). Translation and functional roles of circular RNAs in human cancer. Mol Cancer.

[20] He AT, Liu J, Li F, Yang BB (2021). Targeting circular RNAs as a therapeutic approach: current strategies and challenges. Signal Transduct Target Ther.

[21] Chen B, Dragomir MP, Yang C, Li Q, Horst D, Calin GA (2022). Targeting non-coding RNAs to overcome cancer therapy resistance. Signal Transduct Target Ther.

[22] Kristensen LS, Andersen MS, Stagsted LVW, Ebbesen KK, Hansen TB, Kjems J (2019). The biogenesis, biology and characterization of circular RNAs. Nat Rev Genet.

[23] Zhang J, Cai H, Sun L, Zhan P, Chen M, Zhang F, Ran Y, Wan J (2018). LGR5, a novel functional glioma stem cell marker, promotes EMT by activating the Wnt/beta-catenin pathway and predicts poor survival of glioma patients. J Exp Clin Cancer Res.

[24] Lin S, Li H, Wu B, Shang J, Jiang N, Peng R, Xing B, Xu X, Lu H (2022). TGF-beta1 regulates chondrocyte proliferation and extracellular matrix synthesis via circPhf21a-Vegfa axis in osteoarthritis. Cell Commun Signal.

[25] Li J, Wang X, Chen L, Zhang J, Zhang Y, Ren X, Sun J, Fan X, Fan J, Li T, Tong L, Yi L, Chen L, Liu J, Shang G, Ren X, Zhang H, Yu S, Ming H, Huang Q, Dong J, Zhang C, Yang X (2022). TMEM158 promotes the proliferation and migration of glioma cells via STAT3 signaling in glioblastomas. Cancer Gene Ther.

[26] Srivastava C, Irshad K, Dikshit B, Chattopadhyay P, Sarkar C, Gupta DK, Sinha S, Chosdol K (2018). FAT1 modulates EMT and stemness genes expression in hypoxic glioblastoma. Int J Cancer.

[27] Li X, Guan J, Jiang Z, Cheng S, Hou W, Yao J, Wang Z (2021). Microglial exosome mir-7239-3p promotes glioma progression by regulating circadian genes. Neurosci Bull.

[28] Zhu H, Hu X, Feng S, Gu L, Jian Z, Zou N, Xiong X (2022). Predictive value of PIMREG in the prognosis and response to immune checkpoint blockade of glioma patients. Front Immunol.

[29] Zeng WJ, Zhang L, Cao H, Li D, Zhang H, Xia Z, Peng R (2022). A novel inflammation-related lncRNAs prognostic signature identifies LINC00346 in promoting proliferation, migration, and immune infiltration of glioma. Front Immunol.

[30] Abdalla Y, Luo M, Makila E, Day BW, Voelcker NH, Tong WY (2021). Effectiveness of porous silicon nanoparticle treatment at inhibiting the migration of a heterogeneous glioma cell population. J Nanobiotechnol.

[31] Agudelo-Garcia PA, De Jesus JK, Williams SP, Nowicki MO, Chiocca EA, Liyanarachchi S, Li PK, Lannutti JJ, Johnson JK, Lawler SE, Viapiano MS (2011). Glioma cell migration on three-dimensional nanofiber scaffolds is regulated by substrate topography and abolished by inhibition of STAT3 signaling. Neoplasia.

[32] Yamauchi A, Yamamura M, Katase N, Itadani M, Okada N, Kobiki K, Nakamura M, Yamaguchi Y, Kuribayashi F (2017). Evaluation of pancreatic cancer cell migration with multiple parameters in vitro by using an optical real-time cell mobility assay device. BMC Cancer.

[33] Yang F, Fang E, Mei H, Chen Y, Li H, Li D, Song H, Wang J, Hong M, Xiao W, Wang X, Huang K, Zheng L, Tong Q (2019). Cis-acting circ-CTNNB1 promotes beta-catenin signaling and Cancer progression via DDX3-Mediated transactivation of YY1. Cancer Res.

[34] Lu L, Tian Z, Lu J, Jiang M, Chen J, Guo S, Huang Y (2023). LINC00106/RPS19BP1/p53 axis promotes the proliferation and migration of human prostate cancer cells. PeerJ.

[35] Pei Y, Zhang H, Lu K, Tang X, Li J, Zhang E, Zhang J, Huang Y, Yang Z, Lu Z, Li Y, Zhang H, Dong L (2022). Circular RNA circRNA_0067934 promotes glioma development by modulating the microRNA miR-7/ Wnt/beta-catenin axis. Bioengineered.

[36] Zheng K, Xie H, Wu W, Wen X, Zeng Z, Shi Y (2021). CircRNA PIP5K1A promotes the progression of glioma through upregulation of the TCF12/PI3K/AKT pathway by sponging miR-515-5p. Cancer Cell Int.

[37] Lou J, Hao Y, Lin K, Lyu Y, Chen M, Wang H, Zou D, Jiang X, Wang R, Jin D, Lam EW, Shao S, Liu Q, Yan J, Wang X, Chen P, Zhang B, Jin B (2020). Circular RNA CDR1as disrupts the p53/MDM2 complex to inhibit Gliomagenesis. Mol Cancer.

[38] Pan Z, Zhao R, Li B, Qi Y, Qiu W, Guo Q, Zhang S, Zhao S, Xu H, Li M, Gao Z, Fan Y, Xu J, Wang H, Wang S, Qiu J, Wang Q, Guo X, Deng L, Zhang P, Xue H, Li G (2022). EWSR1-induced circNEIL3 promotes glioma progression and exosome-mediated macrophage immunosuppressive polarization via stabilizing IGF2BP3. Mol Cancer.

[39] Chen J, Wu Y, Luo X, Jin D, Zhou W, Ju Z, Wang D, Meng Q, Wang H, Fu X, Xu J, Song Z. Circular RNA circRHOBTB3 represses metastasis by regulating the HuR-mediated mRNA stability of PTBP1 in colorectal cancer. Volume 11. Theranostics; 2021. pp. 7507–26. 10.7150/thno.59546 PMC8210600 34158864

[40] Wang X, Chen M, Fang L (2021). hsa_circ_0068631 promotes breast cancer progression through c-Myc by binding to EIF4A3. Mol Ther Nucleic Acids.

[41] Liang Y, Wang H, Chen B, Mao Q, Xia W, Zhang T, Song X, Zhang Z, Xu L, Dong G, Jiang F (2021). circDCUN1D4 suppresses tumor metastasis and glycolysis in lung adenocarcinoma by stabilizing TXNIP expression. Mol Ther Nucleic Acids.

[42] Chen G, Long C, Wang S, Wang Z, Chen X, Tang W, He X, Bao Z, Tan B, Zhao J, Xie Y, Li Z, Yang D, Xiao G, Peng S (2022). Circular RNA circStag1 promotes bone regeneration by interacting with HuR. Bone Res.

[43] Ju Z, Lei M, Xuan L, Luo J, Zhou M, Wang Y, Shen L, Skonieczna M, Ivanov DS, Zakaly HMH, Markovic V, Zhou P, Huang R. P53-response circRNA_0006420 aggravates lung cancer radiotherapy resistance by promoting formation of HUR/PTBP1 complex. J Adv Res, (2023). 10.1016/j.jare.2023.07.011 37541584

[44] Geng Y, Wang M, Wu Z, Jia J, Yang T, Yu L (2023). Research progress of circRNA in malignant tumour metabolic reprogramming. RNA Biol.

[45] Nielsen AF, Bindereif A, Bozzoni I, Hanan M, Hansen TB, Irimia M, Kadener S, Kristensen LS, Legnini I, Morlando M, Jarlstad Olesen MT, Pasterkamp RJ, Preibisch S, Rajewsky N, Suenkel C. J. Kjems, Best practice standards for circular RNA research, Nat Methods, 19 (2022) 1208–1220. 10.1038/s41592-022-01487-2 PMC9759028 35618955

[46] Massague J (2008). TGFbeta Cancer Cell.

[47] Zhong T, Zhang W, Guo H, Pan X, Chen X, He Q, Yang B, Ding L (2022). The regulatory and modulatory roles of TRP family channels in malignant tumors and relevant therapeutic strategies. Acta Pharm Sin B.

[48] Birch JL, Coull BJ, Spender LC, Watt C, Willison A, Syed N, Chalmers AJ, Hossain-Ibrahim MK, Inman GJ (2020). Multifaceted transforming growth factor-beta (TGFbeta) signalling in glioblastoma. Cell Signal.

[49] Tzavlaki K, Moustakas A, Signaling TGF-beta. Biomolecules, 10 (2020). 10.3390/biom10030487 PMC7175140 32210029

[50] Morikawa M, Derynck R, Miyazono K. TGF-beta and the TGF-beta family: context-dependent roles in cell and tissue physiology. Cold Spring Harb Perspect Biol, 8 (2016). 10.1101/cshperspect.a021873 PMC4852809 27141051

[51] Zhou M, Yang Z, Wang D, Chen P, Zhang Y (2021). The circular RNA circZFR phosphorylates rb promoting cervical cancer progression by regulating the SSBP1/CDK2/cyclin E1 complex. J Exp Clin Cancer Res.

[52] Qi X, Zhang DH, Wu N, Xiao JH, Wang X, Ma W (2015). ceRNA in cancer: possible functions and clinical implications. J Med Genet.

[53] Ji H, Kim TW, Lee WJ, Jeong SD, Cho YB, Kim HH (2022). Two circPPFIA1s negatively regulate liver metastasis of colon cancer via miR-155-5p/CDX1 and HuR/RAB36. Mol Cancer.

[54] Wu X, Xu L (2022). The RNA-binding protein HuR in human cancer: a friend or foe?. Adv Drug Deliv Rev.

[55] Majumder M, Chakraborty P, Mohan S, Mehrotra S, Palanisamy V (2022). HuR as a molecular target for cancer therapeutics and immune-related disorders. Adv Drug Deliv Rev.

[56] Schultz CW, Preet R, Dhir T, Dixon DA, Brody JR (2020). Understanding and targeting the disease-related RNA binding protein human antigen R (HuR). Wiley Interdiscip Rev RNA.

[57] Chen Y, Yang F, Fang E, Xiao W, Mei H, Li H, Li D, Song H, Wang J, Hong M, Wang X, Huang K, Zheng L, Tong Q (2019). Circular RNA circAGO2 drives cancer progression through facilitating HuR-repressed functions of AGO2-miRNA complexes. Cell Death Differ.

[58] Matsui M, Sakurai F, Elbashir S, Foster DJ, Manoharan M, Corey DR (2010). Activation of LDL receptor expression by small RNAs complementary to a noncoding transcript that overlaps the LDLR promoter. Chem Biol.

[59] He L, Lu Y, Wang P, Zhang J, Yin C, Qu S (2010). Up-regulated expression of type II very low density lipoprotein receptor correlates with cancer metastasis and has a potential link to beta-catenin in different cancers. BMC Cancer.

[60] Chen J, Chen T, Zhu Y, Li Y, Zhang Y, Wang Y, Li X, Xie X, Wang J, Huang M, Sun X, Ke Y (2019). circPTN sponges miR-145-5p/miR-330-5p to promote proliferation and stemness in glioma. J Exp Clin Cancer Res.

