# Supplementary material for: TGF-β-activated circRYK drives glioblastoma progression by increasing VLDLR mRNA expression and stability in a ceRNA- and RBP-dependent manner
Source: J Exp Clin Cancer Res. 2024 Mar 8;43:73. doi: 10.1186/s13046-024-03000-3 (PMC10921701; doi:10.1186/s13046-024-03000-3)

# Three repeats of western blotting

**Figure 2H**

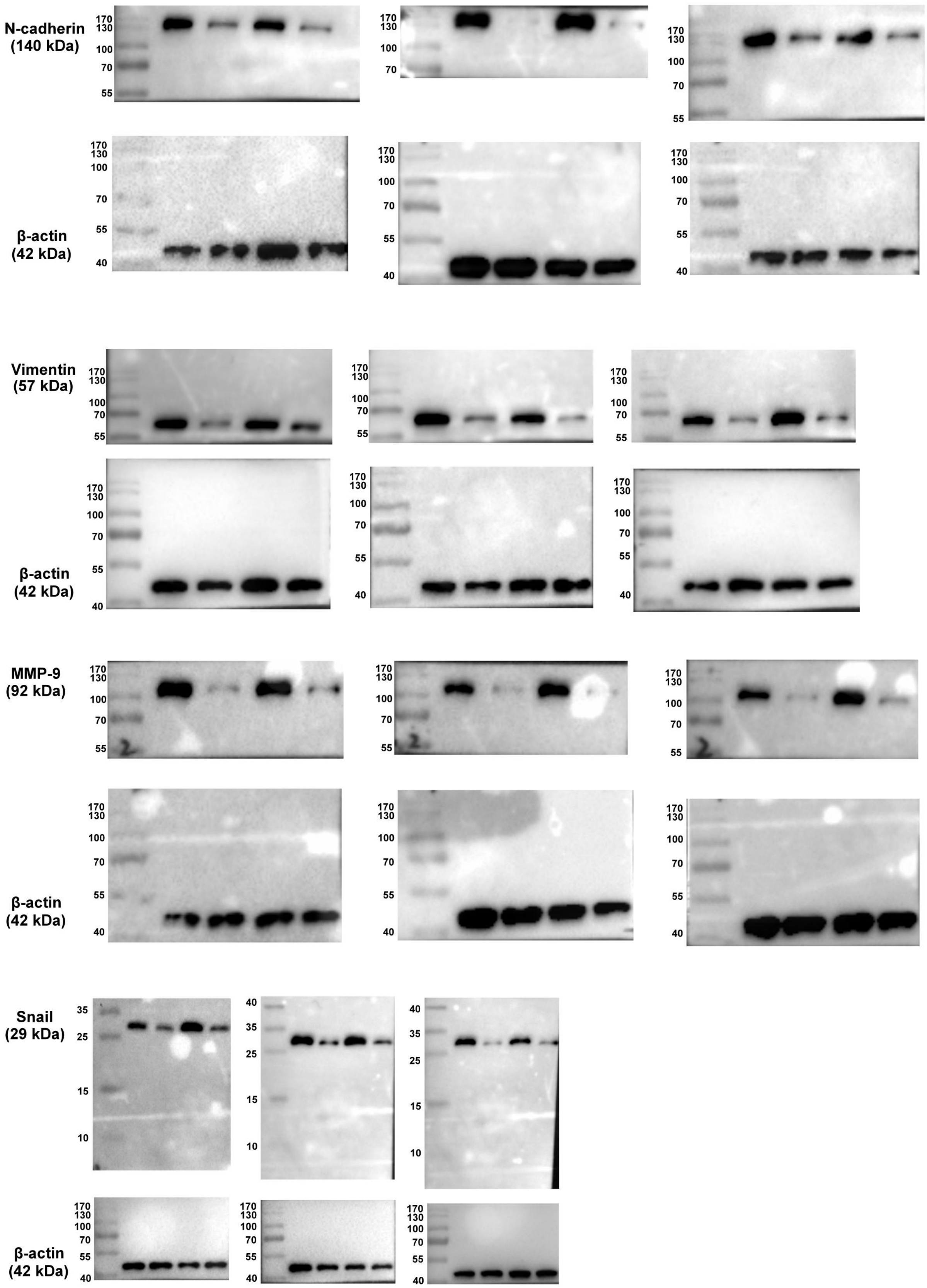

## Figure 2K

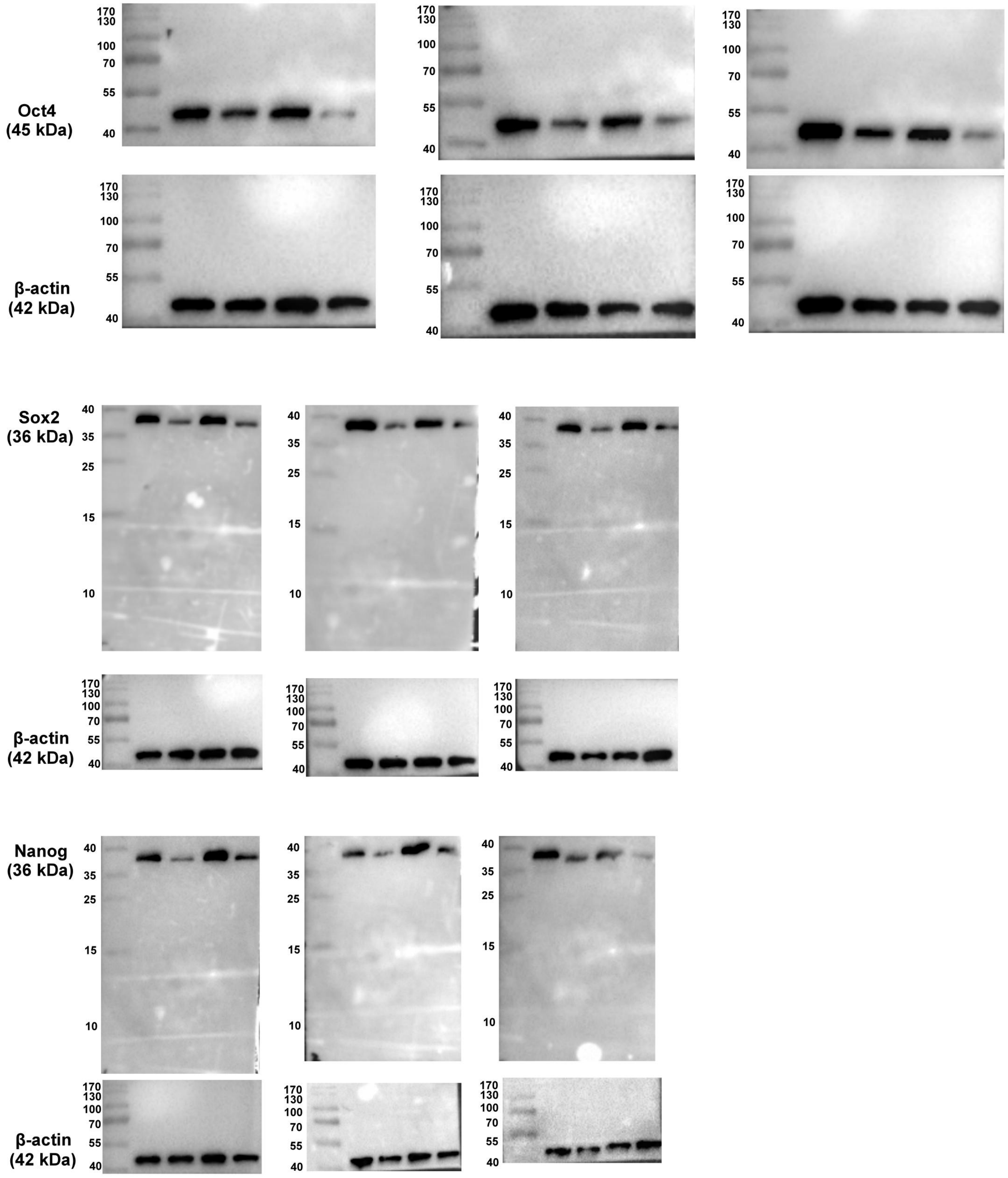
Oct4
(45 kDa)
β-actin
(42 kDa)
Sox2
(36 kDa)
β-actin
(42 kDa)
Nanog
(36 kDa)
β-actin
(42 kDa)
170
130
100
70
55
40
35
25
15
10

**Figure 4D**

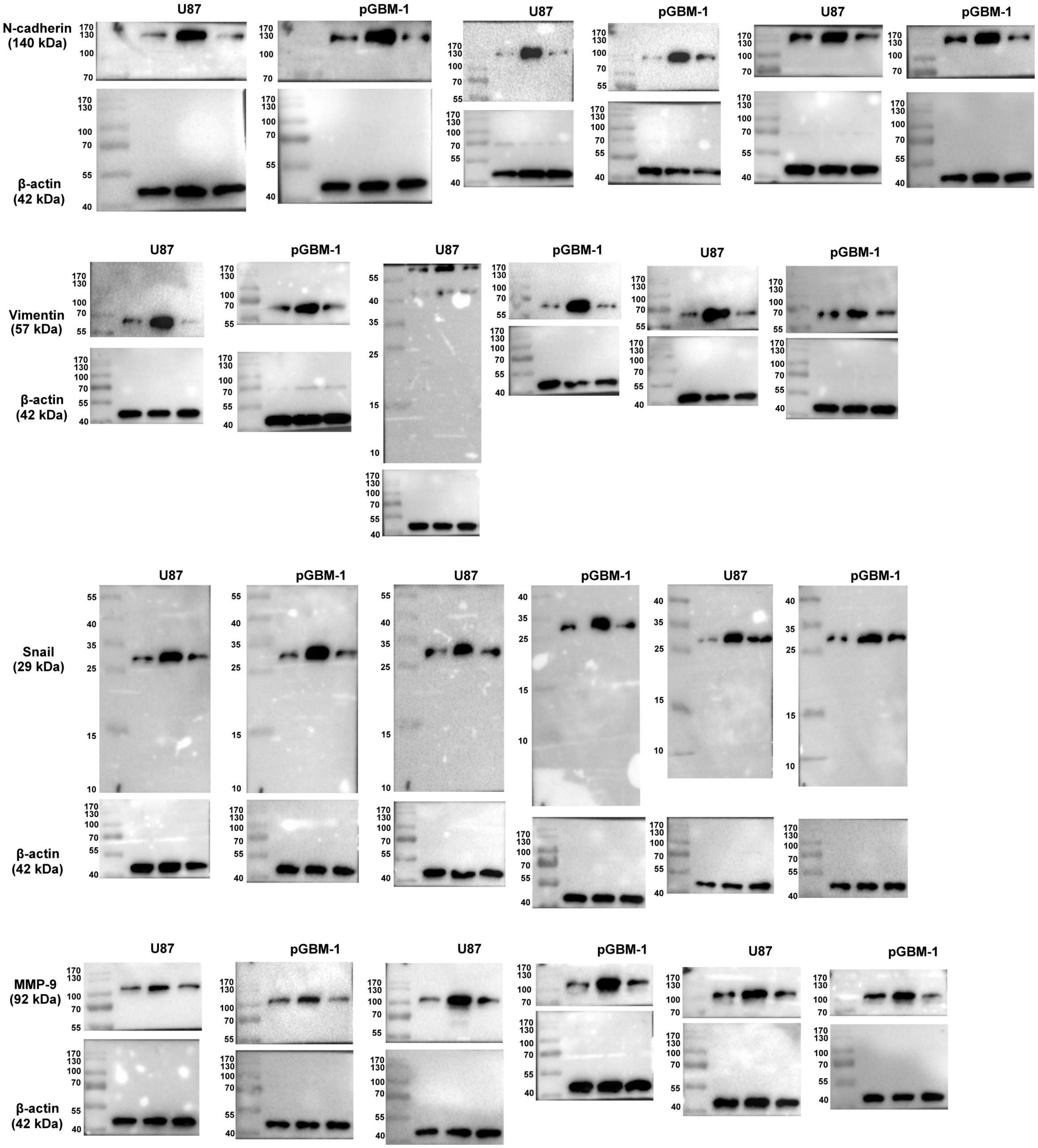

N-cadherin
(140 kDa)
U87
pGBM-1
β-actin
(42 kDa)
Vimentin
(57 kDa)
Snail
(29 kDa)
MMP-9
(92 kDa)

## Figure 4J

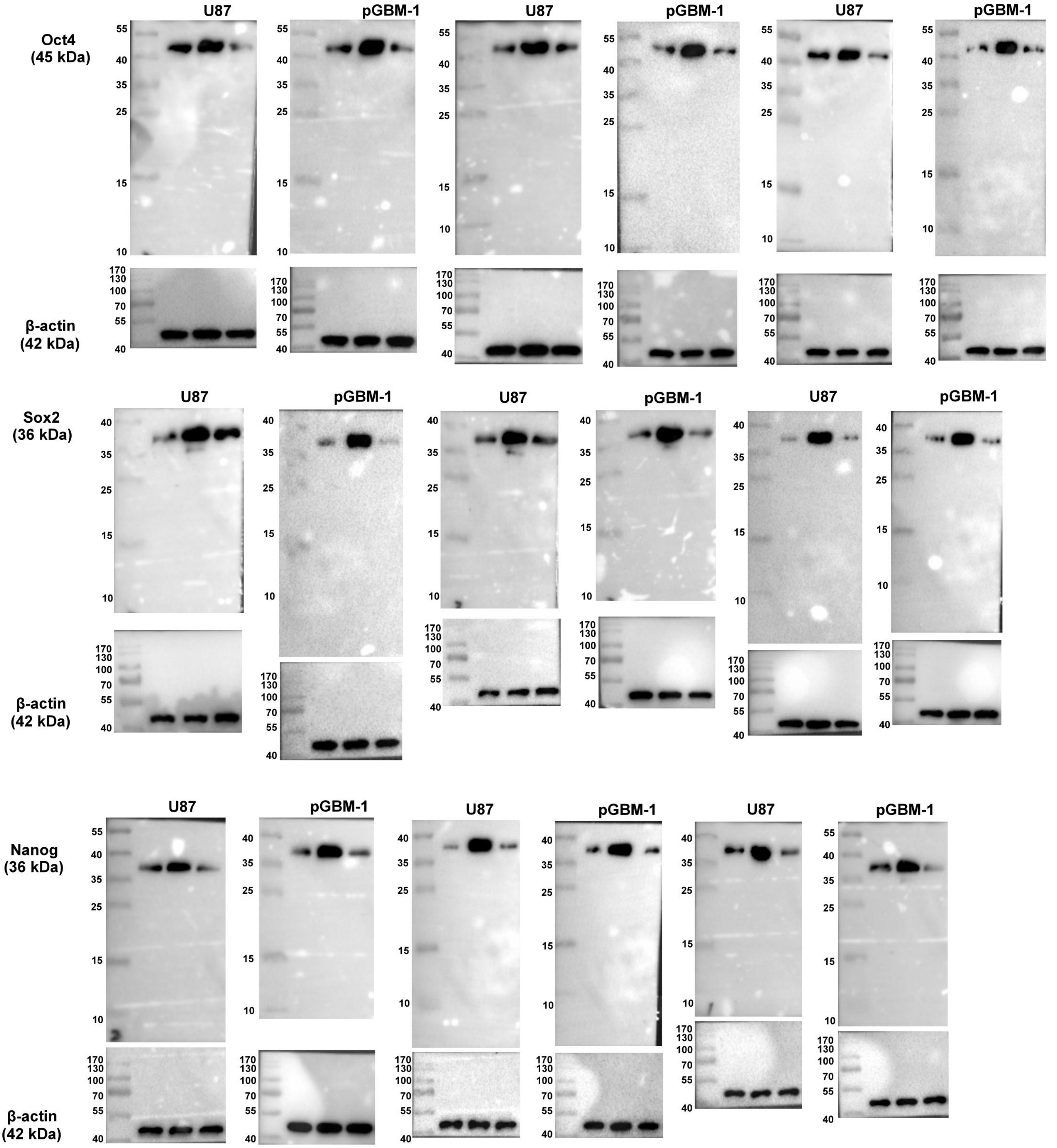

## Figure 6C

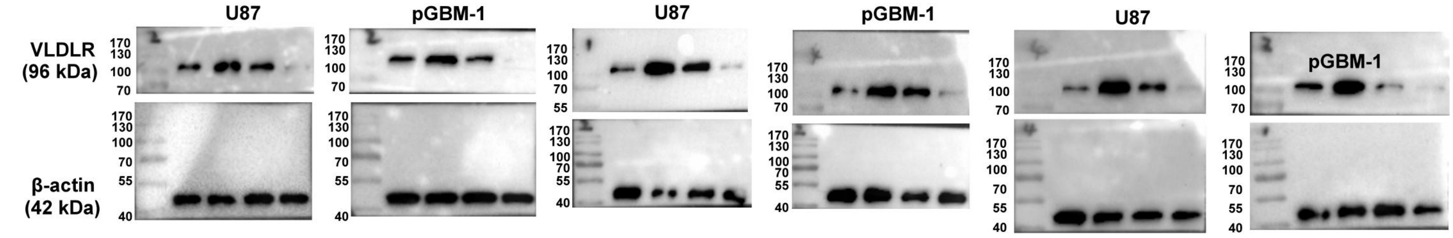


## Figure 6I

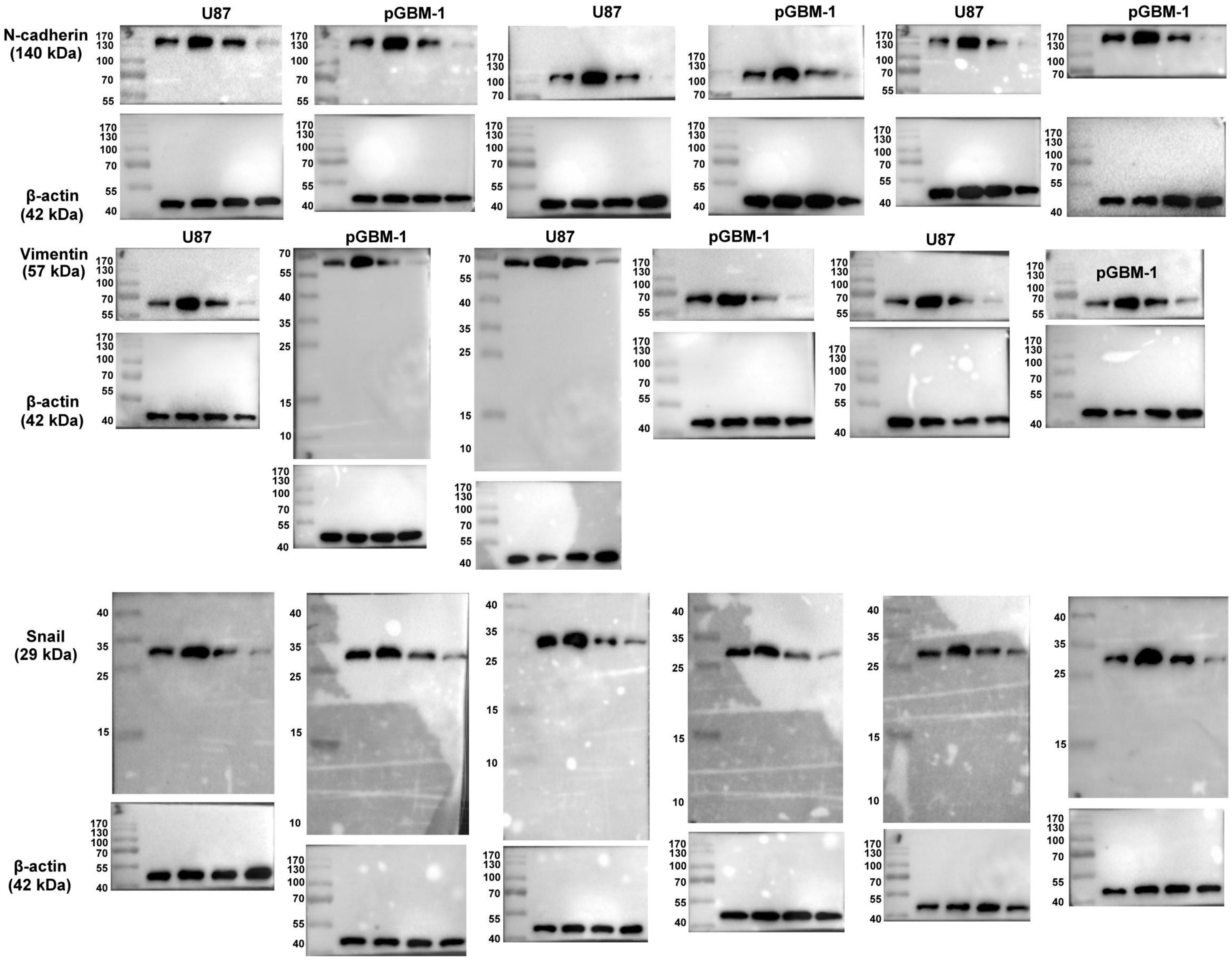

Figure 6L

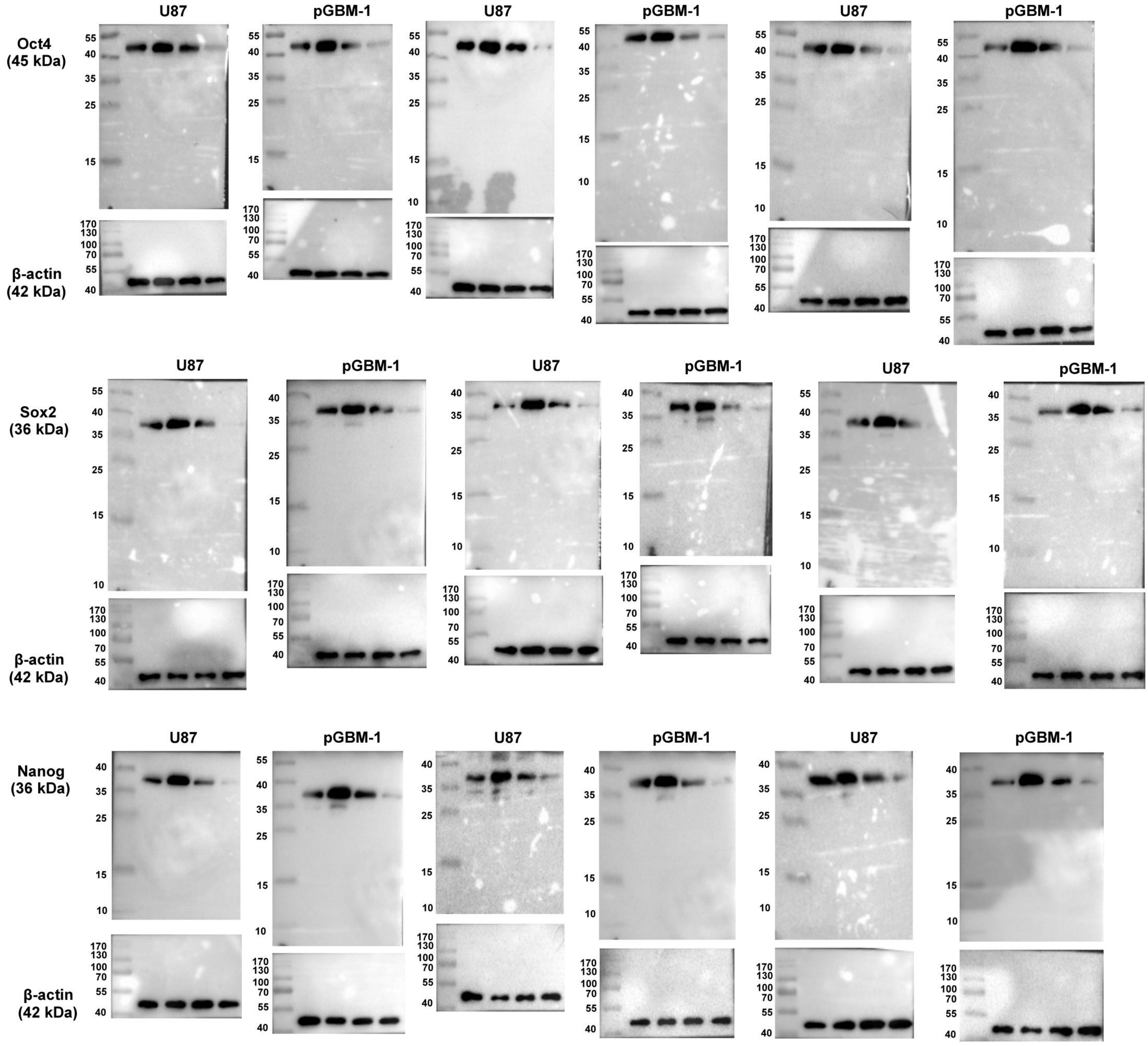

**Figure 7C**

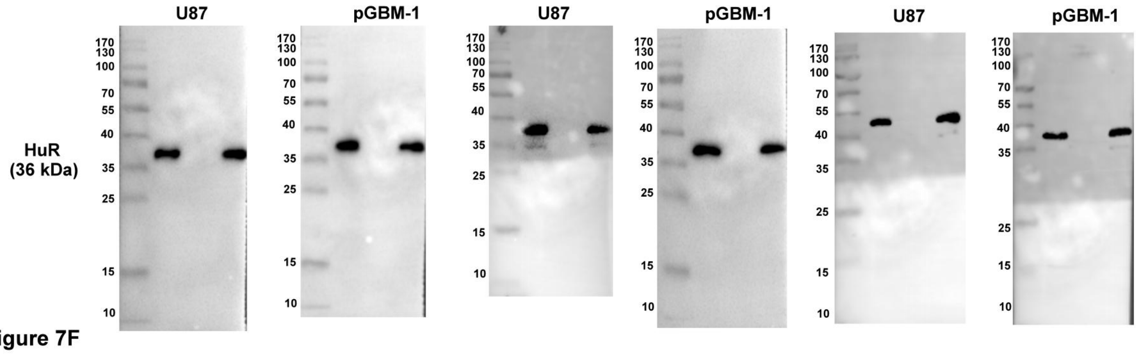


**Figure 7F**

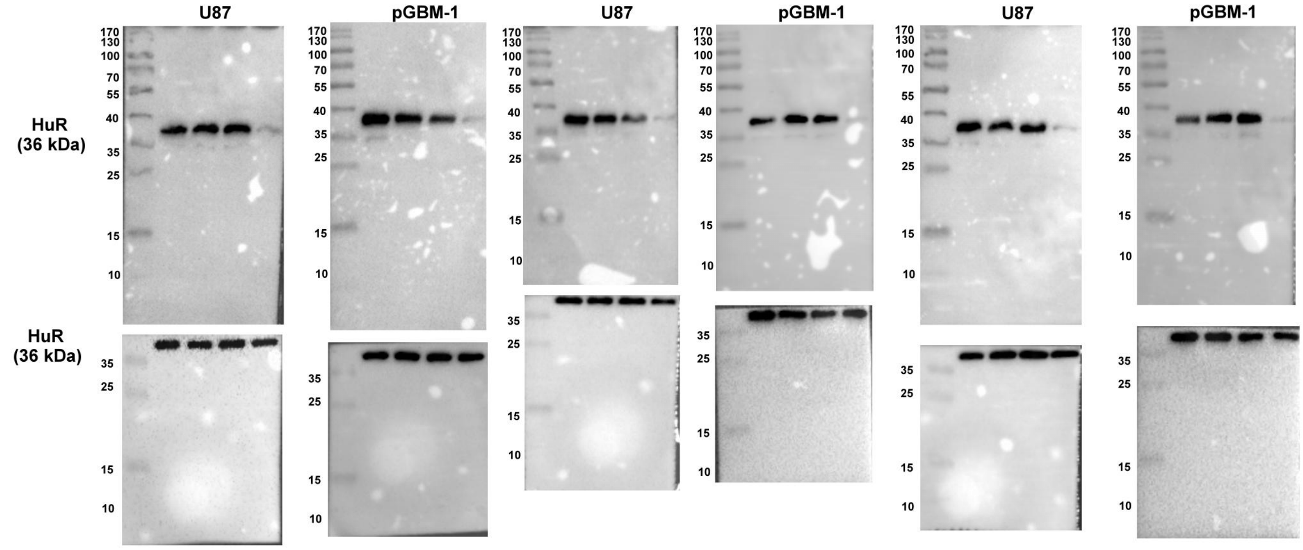


**Figure 7J**

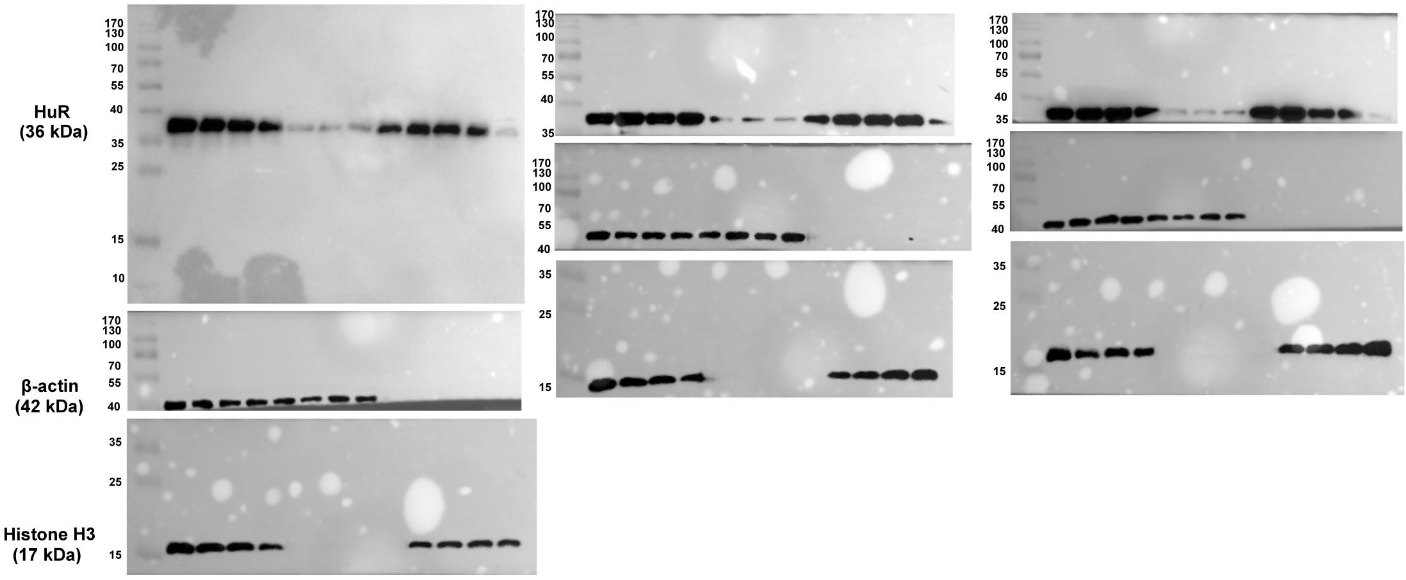

Figure 8F

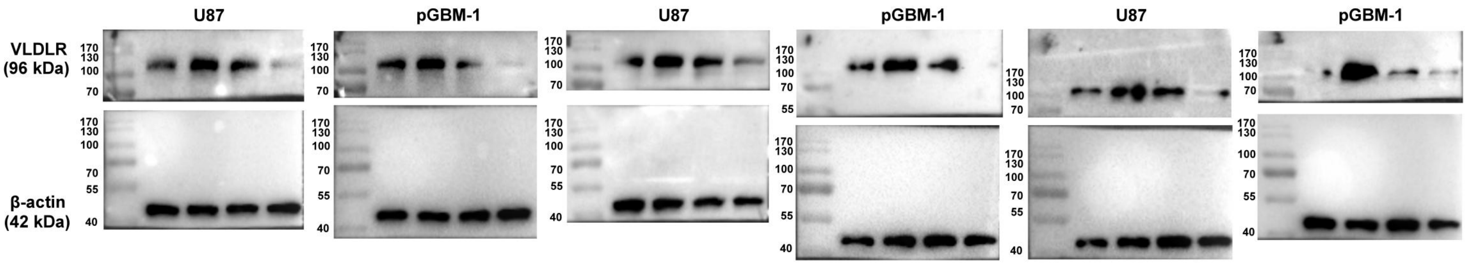


Figure 8H

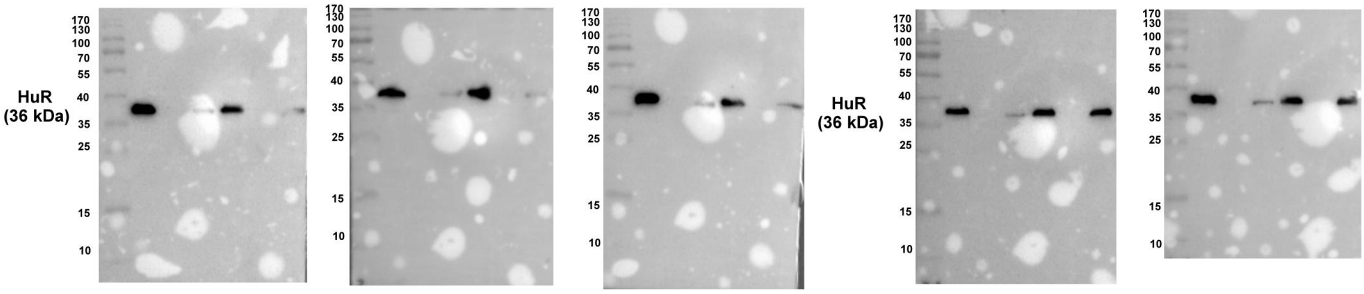


Figure 8L

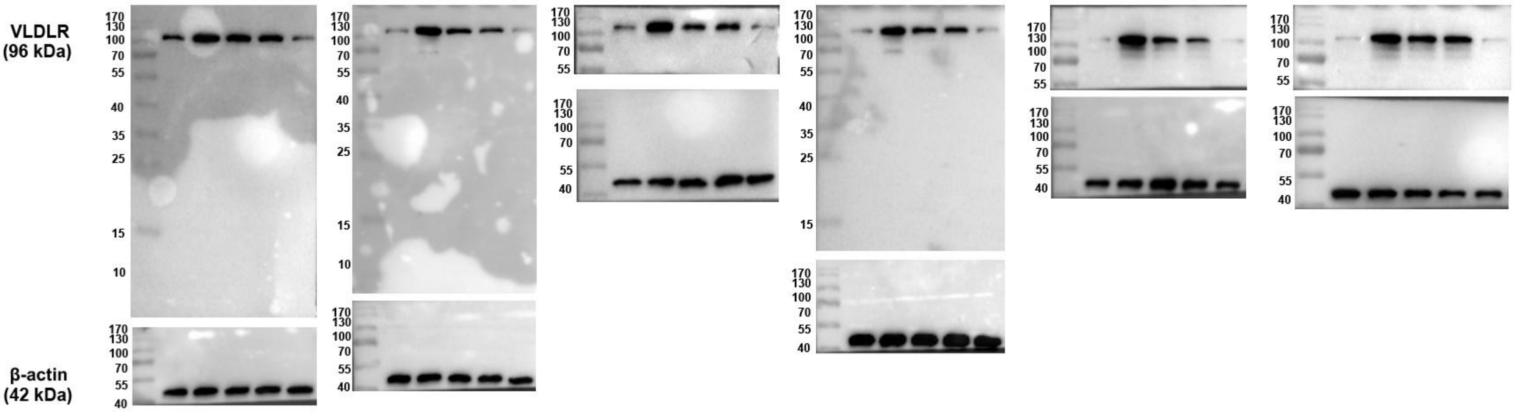

Supplement: Supplementary file 2 — Supplementary Material 2 [file 13046_2024_3000_MOESM2_ESM.pdf]
