# Supplementary material for: TGF-β-activated circRYK drives glioblastoma progression by increasing VLDLR mRNA expression and stability in a ceRNA- and RBP-dependent manner
Source: J Exp Clin Cancer Res. 2024 Mar 8;43:73. doi: 10.1186/s13046-024-03000-3 (PMC10921701; doi:10.1186/s13046-024-03000-3)
Supplement: Supplementary file 4 — Supplementary Material 4 [file 13046_2024_3000_MOESM4_ESM.docx]

**Supplementary figure legends**

**
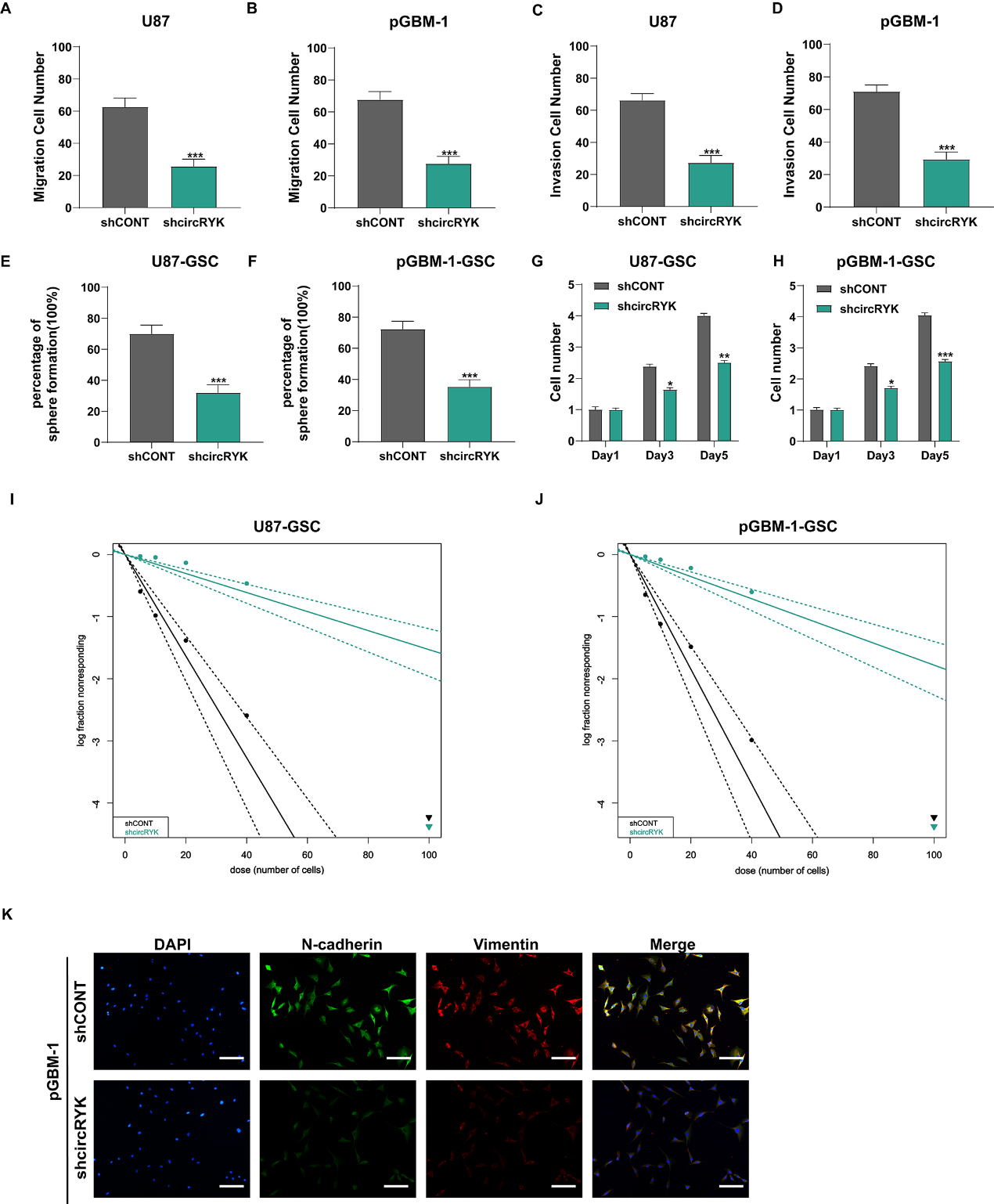
**

**Supplementary Figure 1.** (A-D) Transwell bar chart displaying the data of GBM cells. (E-F) Statistical graph of the clonogenicity assay. (G-H) The proliferation of transfected GBM-GSCs was evaluated utilizing a direct cell count assay. (I-J) The extreme limiting dilution test was performed to define the proliferative ability of GSCs. (K) Immunofluorescence staining was utilized to observe the expression of N-cadherin and Vimentin in treated pGBM-1 cells. Scale bar, 100 μm. Each experiment was performed thrice, and the results are displayed as the mean ± SD (*P<0.05, **P<0.01, ***P<0.001).


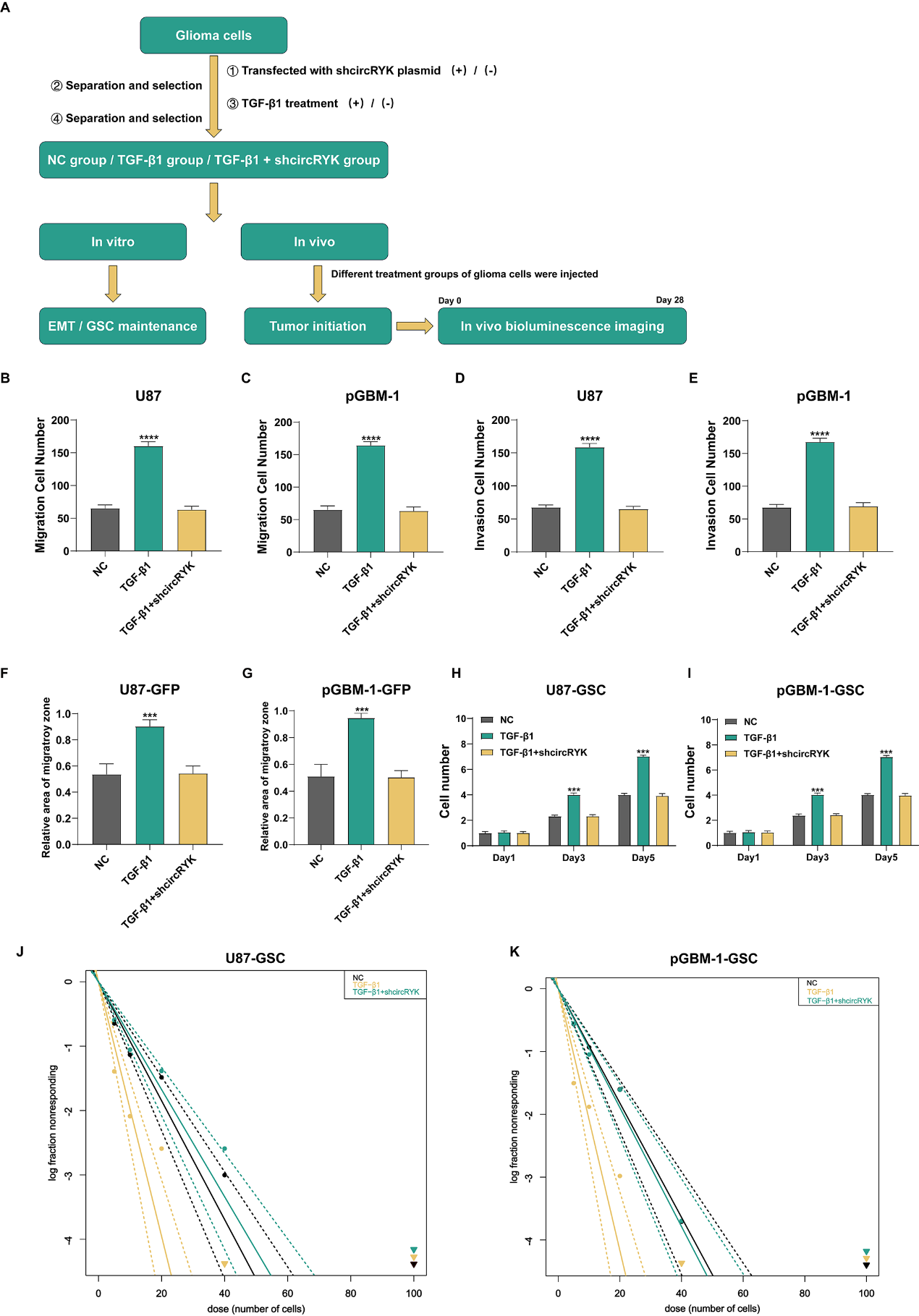


**Supplementary Figure 2.** (A) Schematic illustration of an experiment in which knockdown of circRYK reversed TGF-β1-induced epithelial-mesenchymal transition and GSC maintenance. (B-E) GBM cells were treated with TGF-β1 and shcircRYK alone or in combination to verify the effects on migration and invasion abilities using a Transwell assay. (F-G) The invasion ability of U87-GFP and pGBM-1-GFP cells treated with TGF-β1 and shcircRYK alone or in combination was verified by a three-dimensional spheroid assay. (H-I) Transfected GBM-GSC proliferation was evaluated utilizing a direct cell count assay. (J-K) The extreme limiting dilution test was employed to demarcate the proliferative ability of GSCs treated with TGF-β1 and shcircRYK alone or in combination. Scale bar, 100 μm. Each experiment was executed thrice, and the results are displayed as the mean ± SD (***P<0.001, ****P<0.0001).


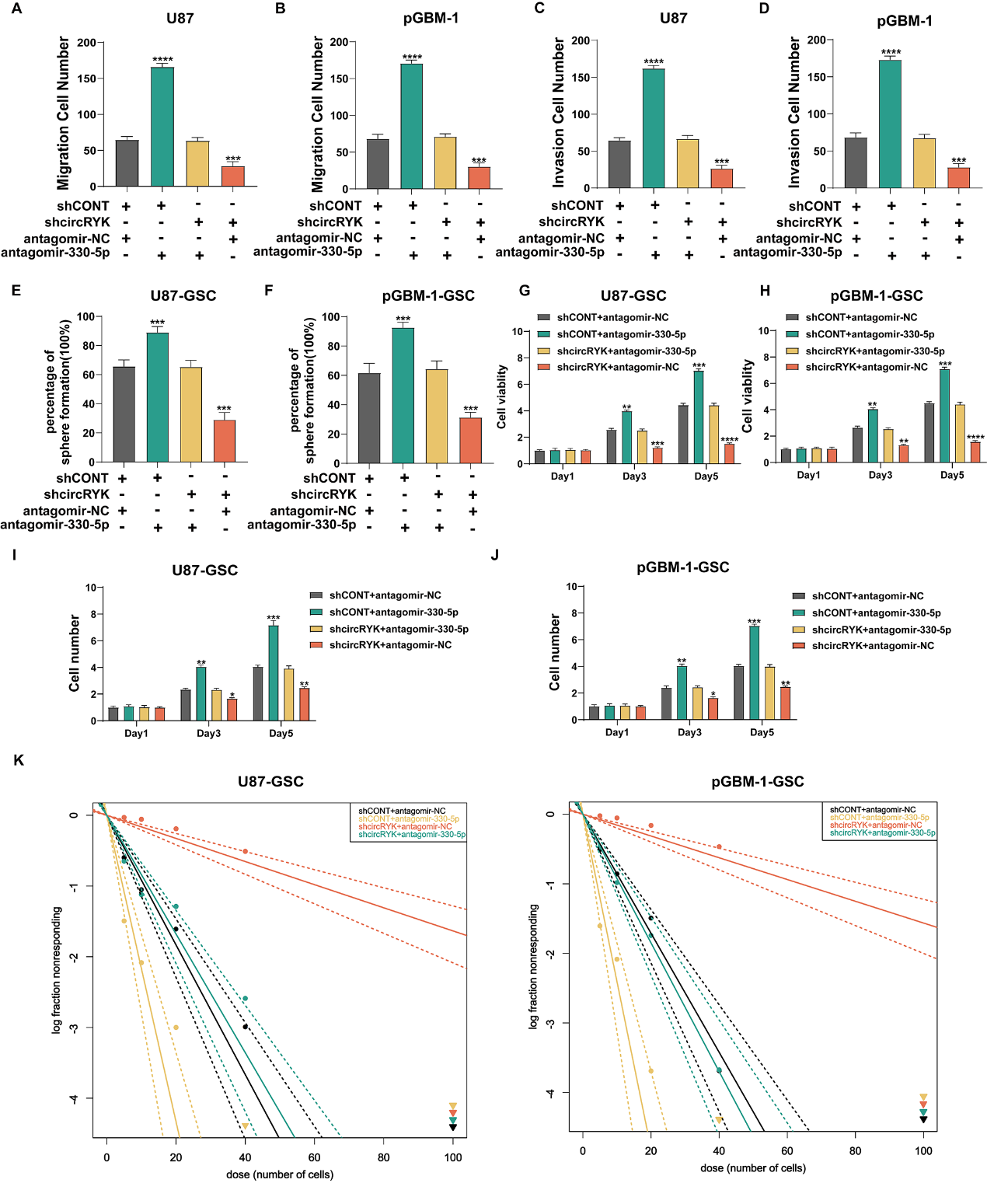


**Supplementary Figure 3.** (A-D) Statistical bar chart comparing GBM cells using the Transwell assay. (E-F) Statistical graph of the clonogenicity assay. (G-J) The proliferation and cellular effects of transfected U87-GSC and pGBM-1-GSC were investigated using the CellTiter-Glo assay and direct cell counts. (K) The extreme limiting dilution test was utilized to evaluate the proliferative capacity of GSCs. Each experiment was performed thrice, and the results are displayed as the mean ± SD (*P<0.05, **P<0.01, ***P<0.001, ****P<0.0001).


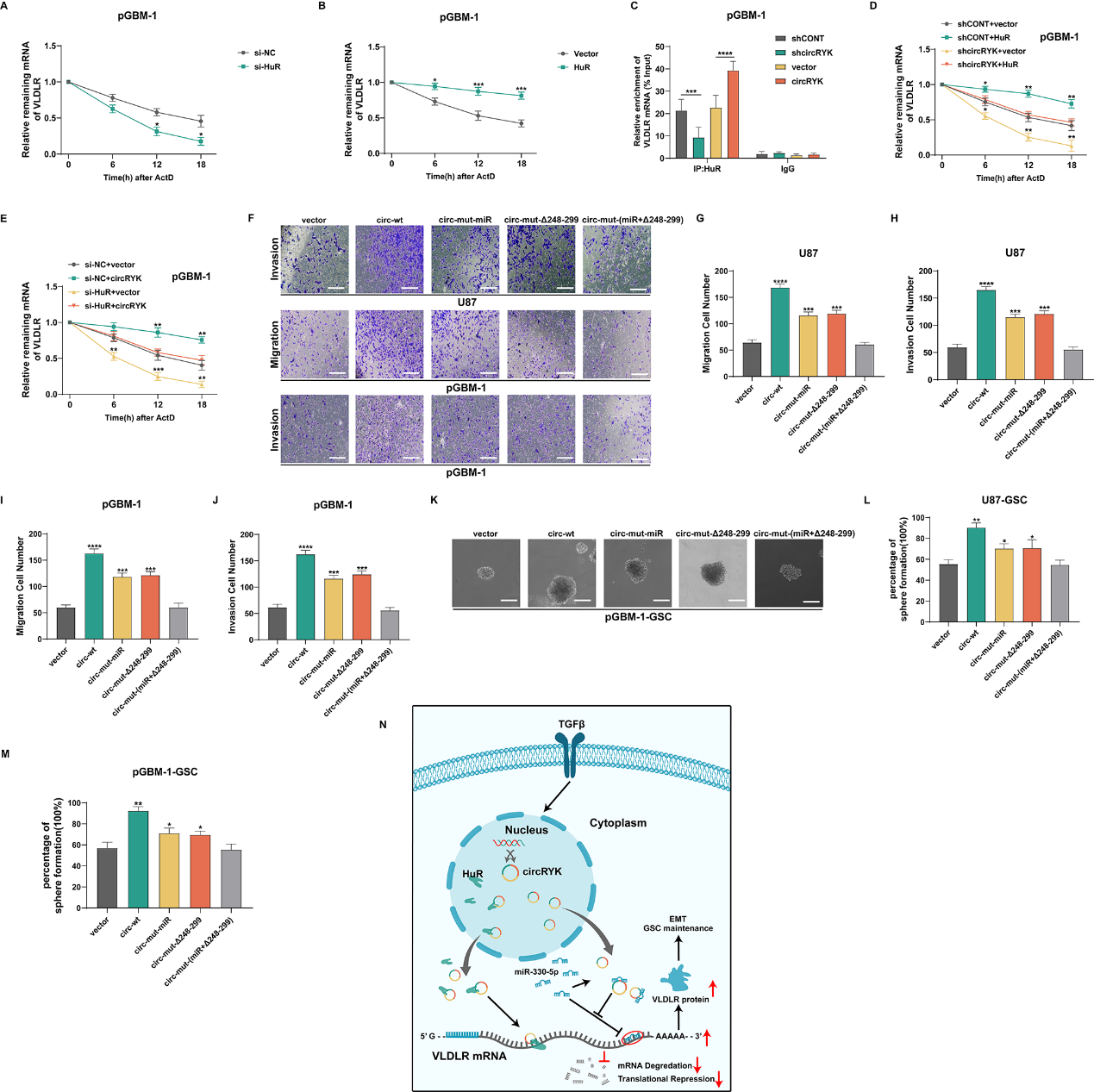


**Supplementary Figure 4.** (A-B) qRT‒PCR was utilized to measure the rates of VLDLR mRNA degradation in HuR-overexpressing or knockdown pGBM-1 cells at various time points. (C) RIP tests showed that when circRYK was elevated or reduced in pGBM-1 cells, HuR and VLDLR mRNA coprecipitated. (D-E) Rate of VLDLR mRNA degradation in pGBM-1 cells transfected with various plasmids or small interferences. (F-M) Transwell and clonogenicity assays were used to verify GBM progression in pGBM-1 and pGBM-1-GSC cells under different treatment conditions. (N) Schematic representation of TGF-β-activated circRYK driving GBM progression. Scale bar, 100 μm. Each experiment was executed thrice, and the results are displayed as the mean ± SD (*P<0.05, **P<0.01, ***P<0.001, ****P<0.0001).


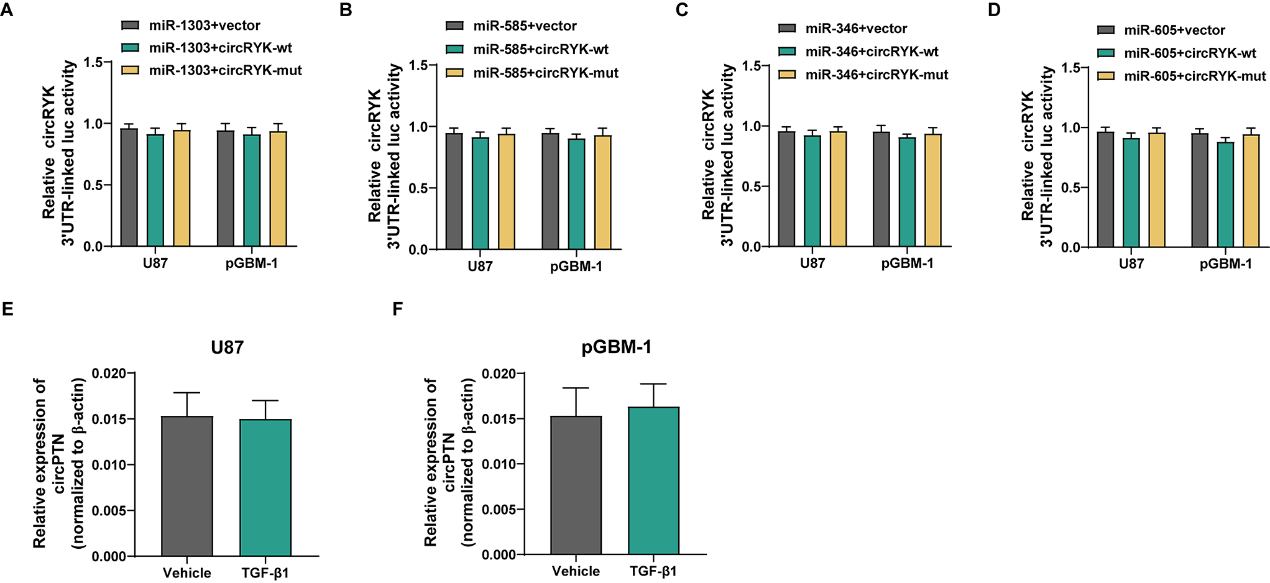


**Supplementary Figure 5.** (A-D) Luciferase reporter assay of circRYK with other miRNAs. (E-F) Expression of circPTN after TGF-β1 treatment. Each experiment was executed thrice, and the results are displayed as the mean ± SD.


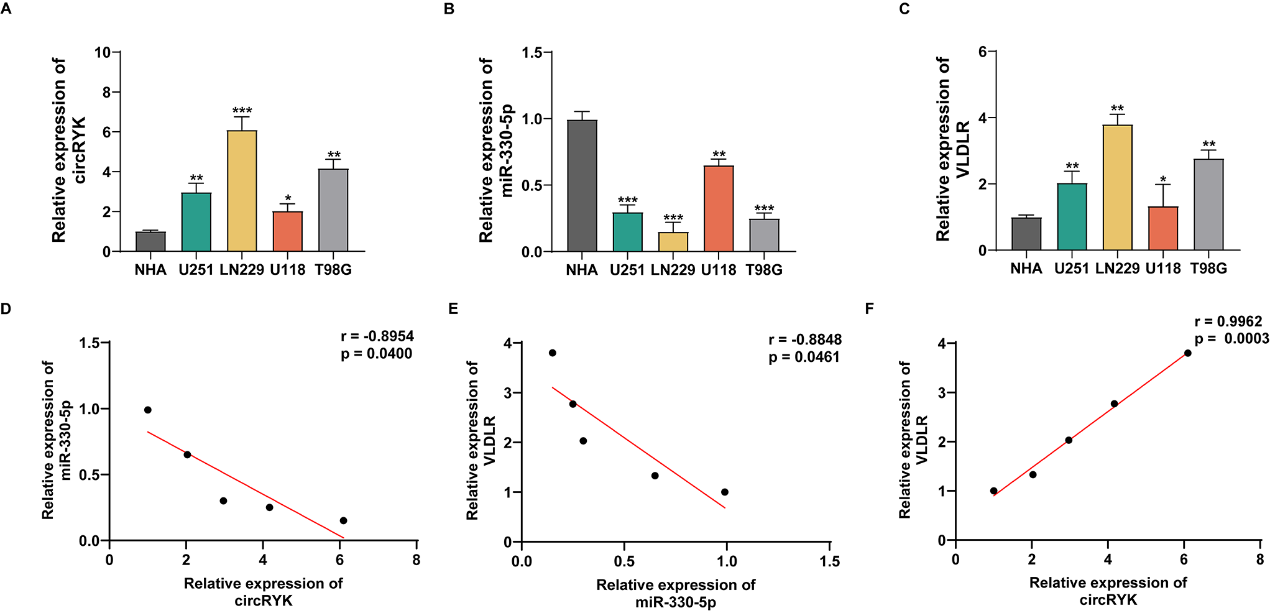


**Supplementary Figure 6.** (A-C) qRT‒PCR was utilized to detect the expression levels of circRYK, miR-330-5p, and VLDLR in NHA, U251, LN229, U118, and T98G cell lines. (D) Using Pearson's correlation, the relationship between the expression levels of circRYK and miR-330-5p was examined. (E) Using Pearson's correlation, the relationship between the expression levels of miR-330-5p and VLDLR was examined. (F) Using Pearson's correlation, the relationship between the expression levels of circRYK and VLDLR was examined. Each experiment was executed thrice, and the results are displayed as the mean ± SD.


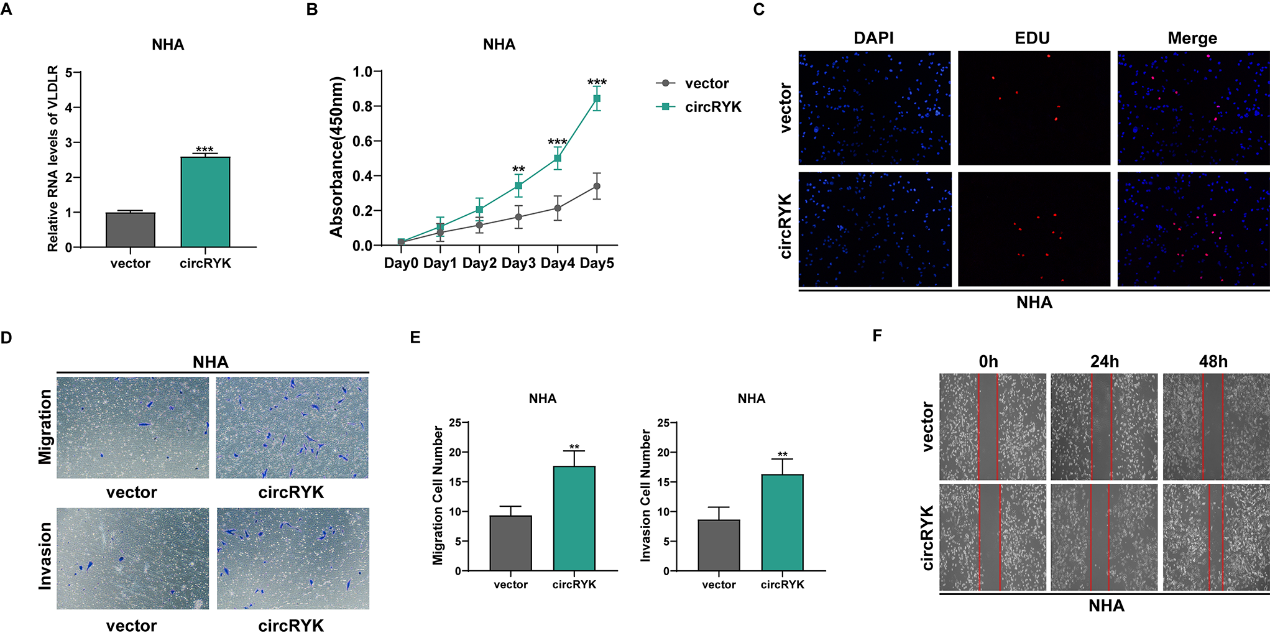


**Supplementary Figure 7.** (A) Expression of VLDLR in NHA after overexpression of circRYK. (B-C) CCK-8 and EDU assays were used to evaluate the changes in the proliferation of NHA after overexpression of circRYK (D-F) Transwell and wound healing assays were used to evaluate the changes in invasion and migration after circRYK overexpression in NHA.

**Supplementary Table 1**

**Patient data and diagnostic standards**

| **Characteristic** | **Glioma patient specimens**  **(n = 40)** | **Normal brain tissues**  **(n = 10)** |
| --- | --- | --- |
| Age, (Mean ± SD) | 52.7 ± 10.8 | 51.9 ± 11.2 |
| Sex |  |  |
| Male, (n, %) | 22 (55.0) | 6 (60.0) |
| Female, (n, %) | 18(45.0) | 4 (40.0) |
| Tumor type, (n, %) |  |  |
| Low-grade glioma | 20 (50.0) | - |
| Anaplastic astrocytoma | 5 (12.5) | - |
| Glioblastoma | 15 (37.5) | - |
| WHO Grade, (n, %) |  |  |
| II | 20 (50.0) | - |
| III | 5 (12.5) | - |
| IV | 15 (37.5) | - |
| Disease specific symptoms, (n, %) |  |  |
| Seizures | 28 (70.0) | - |
| Visual disorder | 16 (40.0) | - |
| Headaches | 22 (55.0) | - |
| Vomit | 7 (17.5) | - |
| Memory decline | 18 (45.0) | - |
| Confusion of consciousness | 6 (15.0) | - |
| Unsteady walking | 4 (10.0) | - |
| Hemiplegia | 9 (22.5) | - |
| Underlying disease, (n, %) |  |  |
| Diabetes | 14 (35.0) | 3 (30.0) |
| High blood pressure | 30 (75.0) | 8 (80.0) |
| Hyperlipemia | 21 (52.5) | 5 (50.0) |
| COPD | 22 (55.0) | 6 (60.0) |
| Coronary heart disease | 10 (25.0) | 3 (30.0) |
| Cerebral apoplexy | 10 (25.0) | 3 (30.0) |
| Thyroid dysfunction | 7 (17.5) | 2 (20.0) |
| Kidney disease | 5 (12.5) | 1 (10.0) |
| Hepatobiliary disease | 9 (22.5) | 2 (20.0) |

**Supplementary Table 2**

**Primer materials for the current study**

| Gene | Primer sequence |
| --- | --- |
| VLDLR mRNA | Forward:5’CGGTCACGACCCATCTCGTTCTGAATCTC -3’  Reverse: 5’- GTGTACGGATATGAAATATACTCACGTTC -3’ |
| hsa_circ_0005768 RYK | Forward: 5’- GAGCTAATATATATGGTAATTCATCG -3’  Reverse: 5’- TTACTGACGTGACTTTCAGAGGTT -3’ |
| RYK mRNA | Forward: 5’- TCACATATGCCTGAATACTT -3’  Reverse: 5’- TACTACTAGTCATACTTGCC -3’ |
| miR-330-5p | Forward: 5’-CGTATCGATATAAGAGATATGA-3’  Reverse: 5’-CGTATCTGTTCATTAATCCTGT-3’ |

**Antibodies for the current study**

| Antibody | Production company |
| --- | --- |
| anti-HuR | abcam, #ab200342 |
| anti-Histone H3 | abcam, # ab1791 |
| anti-Nanog | abcam, #ab109250 |
| anti-VLDLR | abcam, #ab302917 |
| anti-N-cadherin | Cell Signaling Technology, # 13116 |
| anti-Vimentin | Cell Signaling Technology, # 5741 |
| anti-Snail | Cell Signaling Technology, # 3879 |
| anti-Oct4 | Cell Signaling Technology, # 2750 |
| anti-Sox2 | Cell Signaling Technology, #3579 |
| anti-MMP-9 | Cell Signaling Technology, # 13667 |
| anti-β-actin | Cell Signaling Technology, #4967 |
| anti-Rabbit IgG | Cell Signaling Technology, #7074 |
| anti-Mouse IgG | Cell Signaling Technology, #7076 |

**Origin and genetics of pGBM-1**

|  | pGBM-1 |
| --- | --- |
| Gender | Male |
| Age | 51 years old |
| Location | Left frontal lobe |
| Pathological diagnosis | Glioblastoma |
| WHO grade | Ⅳ |
| Ki-67 | 80% (+) |
| IDH1 status | Wild |
| B-raf status | Wild |
| H3F3A status | Wild |
| TERT status | Wild |
| EGFR status | Amplification |
