# Supplementary material for: TGF-β-activated circRYK drives glioblastoma progression by increasing VLDLR mRNA expression and stability in a ceRNA- and RBP-dependent manner
Source: J Exp Clin Cancer Res. 2024 Mar 8;43:73. doi: 10.1186/s13046-024-03000-3 (PMC10921701; doi:10.1186/s13046-024-03000-3)
Supplement: Supplementary file 1 — Supplementary Material 1 [file 13046_2024_3000_MOESM1_ESM.docx]

Dear Editor:

On behalf of my co-authors, I am submitting the enclosed material “**TGF-β-activated circRYK drives glioblastoma progression by increasing VLDLR mRNA expression and stability in a ceRNA- and RBP-dependent manner**” for possible publication in **JOURNAL OF EXPERIMENTAL & CLINICAL CANCER RESEARCH**.

The TGF-β signaling pathway exhibits a strong association with the pathogenesis of glioblastoma (GBM). The primary aim of this study was to analyze the involvement of circular RNAs (circRNAs) in the TGF-β signaling cascade.

Our study found that the expression of circRYK was significantly increased in glioblastoma multiforme (GBM), and this characteristic was highly correlated with an unfavorable outcome. CircRYK plays a crucial role in facilitating epithelial-mesenchymal transition and maintaining glioblastoma stem cell population in glioblastoma multiforme. CircRYK functions by acting as a molecular sponge for miR-330-5p, hence facilitating the upregulation of the oncogenic gene VLDLR. Furthermore, it has been observed that circRYK has the potential to augment the stability of VLDLR mRNA through its interaction with the RNA-binding protein HuR.

Our findings show that TGF-β promotes epithelial-mesenchymal transition and GSC maintenance in GBM through the circRYK-VLDLR axis, which may provide a new therapeutic target for the treatment of GBM.

We appreciate your time and effort on this article, and we look forward to hearing from you.

Respectfully!

Xiupeng Xu

Department of Neurosurgery, The First Affiliated Hospital of Nanjing Medical University, Nanjing, China

Nanjing 210000, China
