# Supplementary material for: TGF-β-activated circRYK drives glioblastoma progression by increasing VLDLR mRNA expression and stability in a ceRNA- and RBP-dependent manner
Source: J Exp Clin Cancer Res. 2024 Mar 8;43:73. doi: 10.1186/s13046-024-03000-3 (PMC10921701; doi:10.1186/s13046-024-03000-3)
Supplement: Supplementary file 3 — Supplementary Material 3 [file 13046_2024_3000_MOESM3_ESM.docx]

Dear Editors and Reviewers:

Thank you and all reviewers for your time and effort in reviewing our paper. We are pleased to note that you have found our research work exciting and pointed out some problems to help us improve the quality of our work. Those comments are all valuable and helpful for revising and improving our paper and the essential guiding significance of our research. We have studied the comments carefully and have made corrections, which we hope to meet with approval. The manuscript was revised following the reviewer's request, and the content and location of the changes were highlighted (with 'tracked changes'/coloured/underlines/highlighted text) in the manuscript. Meanwhile, the English language of our manuscript has been revised by Nature Research Editing Service. I hope the revised manuscript is suitable for publication in **Journal of Experimental & Clinical Cancer Research.**

Yours sincerely,

Xiupeng Xu

The First Affiliated Hospital of Nanjing Medical University

**Responds to Reviewer #1**

Thank you very much for your interest in our manuscript and your valuable suggestions for revision, which will enhance the quality of our manuscript. Our manuscript has been revised according to your advice, and the content and location of the changes are marked in the manuscript.

*1.* *A new language revision should be made, as I have found grammatical errors that could be improved throughout the manuscript.*

**Response to Reviewer #1 Comment No.1:** Thank you very much for being able to point out the shortcomings of our manuscript. Per your suggestion, we have corrected some grammatical errors in our manuscript. Below is our modified editing certificate.

*2. Include in the methodology section the description of TGF- β treatment of the tumoral cells.*

**Response to Reviewer #1 Comment No.2:** We are sorry for this negligence, and We have added a description of TGF-β treatment of the tumoral cells to the Materials and Methods section of the manuscript.

*3.* *In Figure 6 C indicates which cells correspond to each western blot.*

**Response to Reviewer #1 Comment No.3:** We are sorry for our negligence. The cells in Figure 6 C refer to U87 and pGBM-1. We have annotated the cell species corresponding to the western blot in Figure 6 C.

**Figure 6 C**

*4.* *I suggest including a graphic summary describing the role of circRYK in regulating mir-330-5p-VLDRL axis to contribute to glioma malignancy, and its regulation by TGF- β.*

**Response to Reviewer #1 Comment No.4:** We are grateful for your valuable suggestions on our manuscript. Your valuable suggestions are of great help to the improvement of our manuscript. As per your suggestion, we supplemented the graphical summary and put it in the supplementary material.

**Supplementary Figure 4N**

Finally, we are very grateful for the time and effort you have put into our manuscript and provided valuable revision comments. These comments will be of great help in improving the quality of our manuscript. We hope our work can meet your requirements.

**Responds to Reviewer #2**

Our research group would like to thank you for your precious time and effort in reviewing our manuscript. At the same time, thank you for your valuable comments on our manuscript. We very much agree with your modification suggestions and have revised them following your comments.

**Major**

*Major 1.* *The copy numbers of circRYK and miR-330-5p should be determined. It would be important to clarify whether the ratio between these two molecules alone can explain the competitive effect of the endogenous circRYK on miR-330-5p. Alternatively, the involvement of other molecules, which may act as sponges for the same miRNA should be considered and discussed.*

**Response to Reviewer #2 Major Comment No.1:** Thank you very much for your valuable advice. Your comments will improve the quality of our manuscript. Based on your suggestion, we determined the copy numbers of circRYK and miR-330-5p (Fig. 1C and Fig. 5D). Further elucidation of the ratio between these two molecules could explain the competing effects of the endogenous circRYK on miR-330-5p alone. At the same time, we further supplemented experiments to explore other possible targets downstream of circRYK. We extracted the top four miRNAs with high expression except miR-330-5p in Figure 5C and designed the luciferase reporter gene experiment. We found that the luciferase reporter gene assay between circRYK and other miRNAs was not statistically significant (sFig. 5A-D).

According to your suggestion, we further explore whether other molecules act as sponges for miR-330-5p. CircPTN has been reported to promote glioma proliferation and stemness by sponging miR-330-5p[1]. However, our experiments found that the expression of circPTN did not change significantly under the induction of TGF-β (sFig. 5E-F). Although circPTN can contribute to the malignant progression of GBM, we focused more on the altered circRNAs under TGF-β induction affecting the malignant progression of GBM. Therefore, we did not include circPTN within the range of TGF-β-induced circRNAs. As per your suggestion, we have added relevant content to the discussion part of the manuscript. We greatly appreciate your efforts to improve the quality of our manuscript, for which we extend our most sincere respect.

**Responds Figure 1**

*Major 2.* *The authors correctly analyzed circRYK expression across various cell lines, including the pGBM-1 human glioblastoma primary cell line, U87, U118, U251, T98, LN229, and normal human astrocytes (Figure 2A), but then focused on pGBM-1 and U87 cells, which express highest levels of circRYK. It would be important to assess if a linear correlation between the levels of circRYK, miR-330-5p, and VLDLR is maintained also in cell lines with lower levels of circRYK. Have the authors investigated this aspect, and could they comment on the potential correlations across these cell lines?*

**Response to Reviewer #2 Major Comment No.2:** Thank you very much for your valuable comments. According to your suggestion, we further evaluated the linear correlation between circRYK, miR-330-5p, and VLDLR in NHA，U251, LN229, U118, and T98G cell lines. We found the same results with U87 and pGBM-1, thus further demonstrating the importance of the circRYK/miR-330-5p/VLDLR axis in the malignant progression of glioma. Meanwhile, we put the experimental results in Supplementary Figure 6.

**Supplementary Figure 6**

*Major 3.* *It would be interesting to assess whether the overexpression of circRYK per se is sufficient to induce VLDLR expression and eventually alter the normal human astrocytes state, mimicking a glioblastoma phenotype. This result not only would further strengthen the circRYK function as biomarker and promising therapeutical target, but would also highlight its potential primary role in the cause of glioblastoma.*

**Response to Reviewer #2 Major Comment No.3:** Thank you very much for your advice. According to your suggestion, we further evaluated whether circRYK overexpression per se is sufficient to induce VLDLR expression and ultimately alter the normal human astrocyte (NHA) state. We found that the expression level of VLDLR was increased when circRYK was overexpressed in the NHA cell line (sFig. 7A). CCK-8 and EDU experiments showed that overexpression of circRYK caused NHA cell lines to show a tumor-like trend (sFig. 7B-C), further confirmed by Transwell and wound healing experiments (sFig. 7D-F). Further exploration is still needed regarding other potential phenotype of the circRYK-VLDLR axis in NHA and glioma cells. This result not only would further strengthen the circRYK function as biomarker and promising therapeutical target but would also highlight its potential primary role in the cause of glioblastoma.

**Supplementary Figure 7**

**Minor** *Minor 1.* *Page 12 lines 19 and 31:'GBM cells were incubated with 10ng/ml TGF-β1 and shcircRYK plasmid' and 'pGBM-1 cells treated with TGF-β1 alone or in combination with shcircRYK' respectively. It is not clear how the experiments have been performed. Are the cells stable clones transfected with shcircRYK plasmid and then treated or not with TGF-β1? This should be clarified also by providing a schematic representation of the experiment.*

**Response to Reviewer #2 Minor Comment No.1:** Thank you very much for pointing out our shortcomings. In this part of the experiment, we mainly explored that knockdown of circRYK could reverse TGF-β1-induced EMT and GSC maintenance. Therefore, we designed the experimental grouping into three groups: NC group, TGF-β1 treatment group, and TGF-β1 and shcircRYK co-treatment group. We found that treating U87 and pGBM-1 cell lines with TGF-β1 alone induced EMT and GSC maintenance in glioma. However, when we used TGF-β1 stimulation in U87 and pGBM-1 cell lines with knockdown of circRYK, we found that knockdown of circRYK reversed TGF-β1-induced EMT and GSC maintenance. Stable cloned cell lines were screened and isolated for subsequent functional studies *in vitro* and *in vivo*. At the same time, we supplemented the method of TGF-β1 treatment in glioma cell lines in the Materials and Methods section of the manuscript. We apologize for the confusion caused by our inappropriate description in this section. We also extend our sincerest respect to you for reviewing our manuscript. As per your suggestion, the schematic diagram of the experiment is supplemented in Supplementary Figure 2 (sFig. 2A).

**Supplementary Figure 2A**

*Minor 2.* *The authors refer to a 'miR-330-5p inhibitor' at page 14 lines 9-25 and Figure 6. Is it an antagomir? Could they better describe the kind of inhibitor and the concentrations used in the experiments? Such details should be reported also in Materials and Methods section.*

**Response to Reviewer #2 Minor Comment No.2:** We appreciate your valuable advice. We apologize for the confusion caused by our inappropriate description. We want to express our sincerest respect for your efforts in improving the quality of our manuscript. We used miR-330-5p antagomir to inhibit the expression of miR-330-5p in Figure 6. The miR-330-5p antagomir (100 nmol/L) and respective negative control (antagomir-NC) were obtained from GenePharma (Shanghai, China). We have added the relevant introduction to the Materials and Methods section per your suggestions. At the same time, we have corrected the description in the figure.

Finally, I would like to express my appreciation for your advice. Your valuable suggestions will greatly enhance the scientific nature and rigor of our manuscript. I hope our work will meet your requirements.

**Reference**

[1] J. Chen, T. Chen, Y. Zhu, Y. Li, Y. Zhang, Y. Wang, X. Li, X. Xie, J. Wang, M. Huang, X. Sun, Y. Ke, circPTN sponges miR-145-5p/miR-330-5p to promote proliferation and stemness in glioma, J Exp Clin Cancer Res, 38 (2019) 398.
